# Green Space and Health in Mainland China: A Systematic Review

**DOI:** 10.3390/ijerph18189937

**Published:** 2021-09-21

**Authors:** Hania Rahimi-Ardabili, Thomas Astell-Burt, Phi-Yen Nguyen, Juan Zhang, Yu Jiang, Guang-Hui Dong, Xiaoqi Feng

**Affiliations:** 1 Population Wellbeing and Environment Research Lab (PowerLab), School of Population Health, University of New South Wales, Sydney 2052, Australia; hania.rahimi@gmail.com (H.R.-A.); phoebe.nguyen@monash.edu (P.-Y.N.); 2 Centre for Health Informatics, Australian Institute of Health Innovation, Sydney 2113, Australia; 3 Population Wellbeing and Environment Research Lab (PowerLab), School of Health and Society, Faculty of Arts, Social Sciences, and Humanities, University of Wollongong, Wollongong 2522, Australia; thomasab@uow.edu.au; 4 Chinese Center for Disease Control and Prevention, National Institute of Environmental Health, Beijing 102206, China; 5 School of Population Medicine and Public Health, Peking Union Medical College and The Chinese Academy of Medical Sciences, Beijing 100730, China; zhangjuan@sph.pumc.edu.cn (J.Z.); jiangyu@pumc.edu.cn (Y.J.); 6 School of Public Health and Preventive Medicine, Monash University Victoria, Clayton 3004, Australia; 7 Department of Environmental and Occupational Health, School of Public Health, Sun Yat-sen University, Guangzhou 510080, China; donggh5@mail.sysu.edu.cn

**Keywords:** green space, mainland China, health outcomes, systematic review

## Abstract

Non-communicable diseases (NCDs) have become a major cause of premature mortality and disabilities in China due to factors concomitant with rapid economic growth and urbanisation over three decades. Promoting green space might be a valuable strategy to help improve population health in China, as well as a range of co-benefits (e.g., increasing resilience to climate change). No systematic review has so far determined the degree of association between green space and health outcomes in China. This review was conducted to address this gap. Five electronic databases were searched using search terms on green space, health, and China. The review of 83 publications that met eligibility criteria reports associations indicative of various health benefits from more green space, including mental health, general health, healthier weight status and anthropometry, and more favorable cardiometabolic and cerebrovascular outcomes. There was insufficient evidence to draw firm conclusions on mortality, birth outcomes, and cognitive function, and findings on respiratory and infectious outcomes were inconsistent and limited. Future work needs to examine the health benefits of particular types and qualities of green spaces, as well as to take advantage of (quasi-)experimental designs to test greening interventions within the context of China’s rapid urbanization and economic growth.

## 1. Introduction

China has experienced a tremendous epidemiological and demographic transition in the past 30 years, driven in part by rapid economic growth and urbanisation. These changes have markedly shifted the country’s leading causes of death [1]. Non-communicable diseases (NCDs) such as cardiovascular diseases (CVDs), cancer, and mental health disorders have become a major cause of premature mortality and disabilities in China [2]. High blood pressure, unhealthy behaviours, and air pollution were among the four leading risk factors contributing to deaths and disabilities. Research shows that levels of risk factors are rising, with the highest increase for the risk factors of body mass index (BMI) and ambient particulate matter (PM) in the last few decades [2]. The National Health Commission of the People’s Republic of China plans to support the WHO’s aim of reducing premature mortality from NCDs by one-third by 2030 compared with the current level. Thus, China aims to adopt health policies or strategies for improving the overall health of the Chinese population over the next 15 years, with particular attention to NCD prevention and control.

Urban greening might be a valuable strategy to assist the Chinese government in reaching their goal. The term green space is typically defined as open, undeveloped land with natural vegetation, although it also exists in many other forms such as urban parks and public open spaces as well as street trees and greenery [3]. A higher quantity of nearby green space has been reported to be beneficial for health in high-income ‘Western’ countries by reducing the risks of NCDs and associated risk factors [4,5,6]. Mechanisms underlying the benefits of green space can be attributed to its three domain pathways [7]. These are: (1) harm mitigation, e.g., of air pollution and excess heat; (2) restoration of depleted capacities, e.g., psychological and immunological; and (3) building capacities for better health, e.g., through encouraging physical activity and social connection.

While there is a substantial amount of evidence supporting the health benefits of green space, local evidence is needed due to China’s relatively unique situation. China’s urbanisation has an unprecedented rate. This is the largest scale internal migration worldwide [8]. Urban growth leads to a lack of access to greenery and poor air quality [9]. Air quality in China has been reported to be generally poor, although some improvements have been noted in recent years [10,11]. Despite the government’s effort in the last two decades to construct or preserve green space, after the initial increase in green space coverage [12], China later faced the loss of public green spaces with the increasing pace of urbanisation [13]. China is also outstanding regarding its fast pace of economic growth. Thanks to the past few decades’ economic advancements, China is now the world’s second-largest economy [14]. In addition, China has the largest population and is undergoing rapid population ageing. Unlike many high-income countries like the US and Australia, people in Chinese cities tend to live in very high-density apartments. China has the largest number of older people worldwide [15], and the rate is higher than the global average [16]. This could be particularly important as many oldest people are dependent on the immediate environment due to mobility limitations [17].

Anecdotal observation indicates that China has been the setting of many studies of green space and health, especially in recent years. However, no systematic review has attempted to critically synthesise evidence of association between green space and health outcomes in China to date. Our aim was to address this gap. We critically synthesised the emerging evidence on green space and health in mainland China. In particular, we took into account trends in the types of studies and measurement of variables over time, as well as sources of geographical heterogeneity given uneven development across China’s vast terrain. Finally, in understanding the work done thus far, we sought to identify avenues for future research.

## 2. Materials and Methods

This review was conducted according to the Preferred Reporting Items for Systematic Review and Meta-Analyses (PRISMA) guidelines for systematic reviews [18].

### 2.1. Study Selection

Articles were included if they evaluated the effects of green space on health outcomes in China, were conducted in humans, were peer-reviewed, and were published in English. Table 1 outlines detailed inclusion and exclusion criteria.

### 2.2. Search Strategies

The following electronic databases were searched on 25 Nov 2020: MEDLINE, EMBASE, Scopus, Cumulative Index to Nursing and Allied Health Literature (CINAHL), and PsycINFO. No restriction on publication date or publication status was used. Search terms were partially adapted from previous systematic literature reviews on green space and health outcomes overall [4,19,20], obesity and physical activity [21,22], birth outcomes [5], mental health [23,24,25], puberty timing [26], and menopause [27]. We used keyword combinations of ‘green space’ and ‘health’ and ‘China’ searched for in titles and abstracts. In addition, the investigators consulted a librarian to ensure the search strategy was comprehensive. The current review is a part of a larger systematic review project, including countries located in the Western Pacific Region and South-East Asia Regions. Thus, the search strategy included a list of those countries. Studies conducted in the Western Pacific Region and South-East Asia Regions were excluded in the title/abstract screening for the purpose of the current review. See supplementary Table S1 for the complete search strategy for each database.

Study selection was completed via a two-step screening process using Covidence software (Covidence systematic review software, Veritas Health Innovation, Melbourne, Australia). After deduplication of search results using the Endnote reference manager, two reviewers (H.R.-A. and P.N.) independently screened the title/abstract of all articles to identify eligible articles. Any disagreements were resolved by a third reviewer (T.A.B.). Full texts were then sourced for the articles identified from the title/abstract screening stage. One reviewer (H.R.-A.) verified each of these full-text articles to confirm their relevance for inclusion in the review. When a reviewer was unclear on whether to ‘include’ or ‘exclude’, a discussion between all investigators took place to reach a decision. In addition, to assess inter-rater reliability, 10% of articles’ full texts was reviewed by a second reviewer (P.N.), with any disagreements resolved by a third reviewer (X.F.). The reference lists of the relevant articles were also reviewed by one reviewer (H.R.-A.) to identify any other eligible studies that were missed in the initial search process.

### 2.3. Data Extraction

One author (H.R.-A.) extracted and synthesised data from the included articles into an Excel sheet.

The extracted data included author information, year of publication, study area, study design, population, and sample size. In addition, we collected and synthesised data on environmental focus, methods used to measure green space and health outcomes, statistical analysis, covariates adjusted, main results, and mediating and moderating factors if assessed. Associations between green space and health indicators are presented as positively associated, null, and negatively associated along with their level of statistical significance for every individual outcome.

### 2.4. Quality Assessment

The National Heart, Lung, and Blood Institute quality assessment tools were used to evaluate the quality of all included articles [28]. The tools include 12–14 criteria depending on the study design, referring to several aspects of studies, including sample population, the objectiveness of exposure and outcome variables, and randomisation and blinding. For each item in the list, three options for answers are suggested, which are ‘Yes’, ‘No’, or ‘Other’ (NR, NA). If the criteria were met (Yes) it was assigned the value of 1, whereas if the criterion was not met (No), was not reported (NR), or was not applicable (NA), 0 points were assigned. Finally, the overall score was calculated by dividing the sum of the positive scores by the total number of applicable questions. Scores below 50, between 50 and 74, or above 75 meant the articles were regarded as low, fair, and high quality, respectively. The same classification was used previously [5,29]. One reviewer (HR-A) conducted the quality assessment independently with 10% reviewed by a second reviewer (PN). Disagreements were discussed to reach an agreement.

## 3. Results

**Figure 1:**
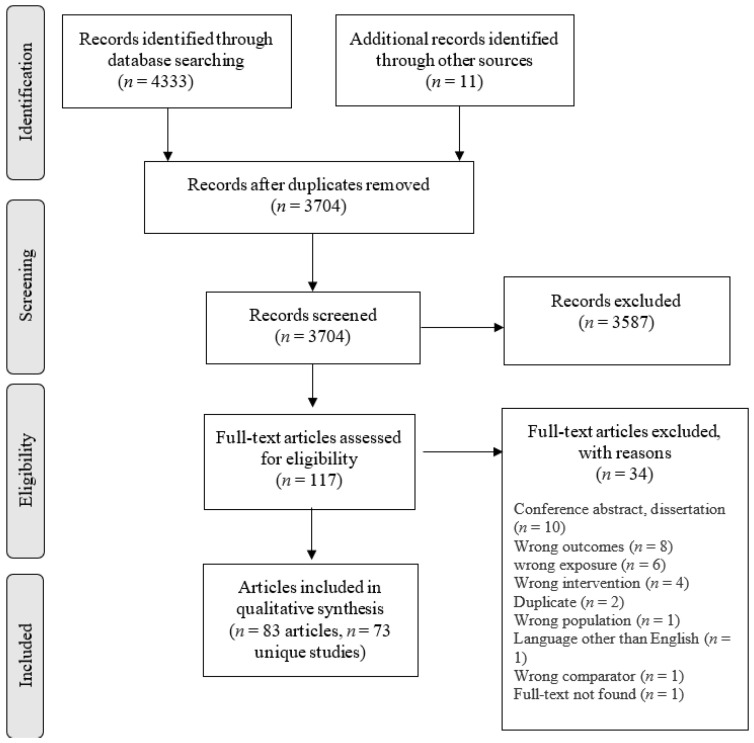
PRISMA flow diagram.

The initial searches resulted in 4333 records. After removing duplicates, 3693 articles remained for the title/abstract screening. Full texts of 130 articles were reviewed, and 83 articles were selected for this systematic review. Of 83 articles, two contained two studies each (i.e., each had two different sets of samples and study designs). Some articles described different outcomes based on the same study (i.e., analysing data from the same sample) in six instances as follows: two articles described one study on three occasions, three articles on two occasions, and six articles on one occasion. This led to 73 unique studies. In the current review, the unit of data synthesis is ‘study’ (i.e., 73 studies) unless otherwise specified. Of these studies, eight have analysed some data from the pool of the Chinese Longitudinal Heathy Longevity Survey (CLHLS) cohorts. For a summary of the search process, see the PRISMA flow diagram in Figure 1.

### 3.1. Study Characteristics

**Figure 2:**
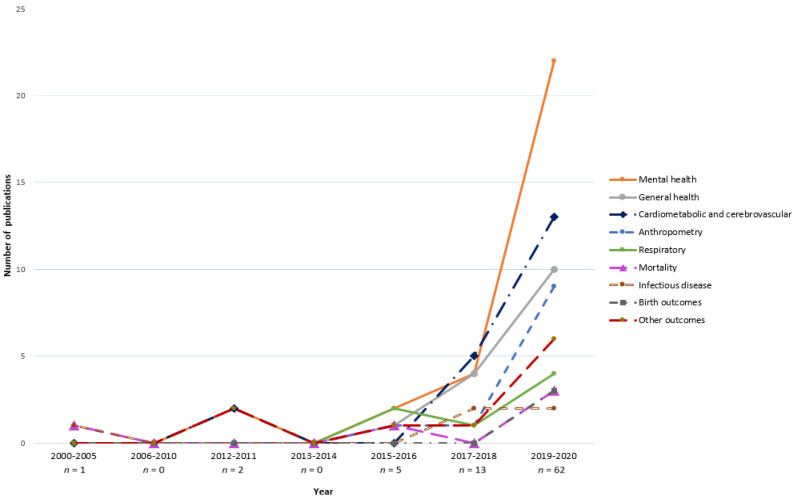
Publication trend by year and health outcomes. Some studies measured more than one outcome. *n*, total number of publications.

Studies were published from 2002 to 2021, with more than 90% (*n* = 75 articles) of them being published in the last four years from 2017 up to 2020 (Figure 2). Over half of the studies (52%) were conducted in highly urbanised (urbanisation rate of 70% and over) areas of the country (i.e., Shanghai, Beijing, Tianjin, Guangdong, Jiangsu, Zhejiang) [30], whereas one-third (*n* = 21) were conducted with a nationwide sample.

**Figure 3:**
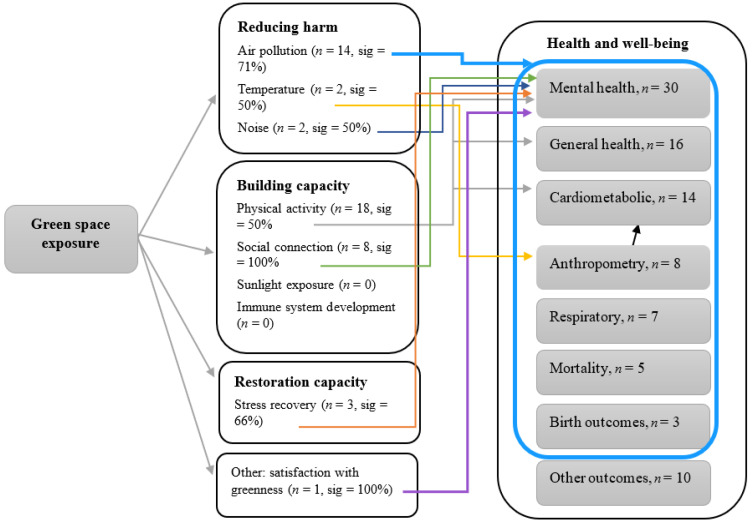
Pathways linking greenspace to health outcomes examined in the 73 studies in China. Arrows link particular pathways with health outcomes, with a single arrow from air pollution associated with all health outcomes contained within the blue circle. *n*, number of studies; sig, proportions of studies that found a significant effect of the mediator.

Study samples were mostly a balanced proportion of men and women. A few studies were conducted in women [31,32,33,34] or men [35,36] only. A cross-sectional design was the most frequently utilised design (*n* = 42); other designs used were cohort (*n* = 9), randomised controlled trial (RCT; *n* = 9), ecological (*n* = 7), case–control (*n* = 3), before–after (*n* = 2), and quasi-experimental (*n* = 1). Studies were mostly rated as fair quality, and about 20% (*n* = 16) and 19% (*n* = 14) were rated as high and low quality, respectively (Table S2). A wide range of health outcomes were measured in the included studies. Health outcomes were grouped into seven categories of (1) mental health, including all psychological and mental health-related outcomes such as restoration effects and mood states (*n* = 30), (2) generic health, including an overall assessment of physical health or a combination of physical and mental health (*n* = 16), (3) cardiometabolic and cerebrovascular outcomes (*n* = 15), (4) anthropometry (*n* = 8), (5) respiratory diseases (*n* = 7), (6) mortality (*n* = 5), (7) infectious diseases (*n* = 4), and (8) birth outcomes (*n* = 3). Any other outcomes such as stress markers and sleep quality that do not fit into the following categories are discussed as ‘other’ outcomes. Details about the studies characteristics are further provided within each subheading of health outcomes. Figure 3 illustrates an outline of the current review’s findings. Study characteristics and results are summarised in Table S3 tabulated by health outcome categories.

### 3.2. Green Space Measurement

Various methods were used for measuring green space exposure. Of them, normalised difference vegetation index (NDVI) (*n* = 30), green space coverage (*n* = 13), proximity to green space (*n* = 10), green space per capita (*n* = 12), and street view (*n* = 5) were the most common approaches. Table 2 represents the distribution of studies across different tools used for assessing green space exposure for each health outcome category. All studies except for eight [37,38,39,40,41,42,43,44], used an objective measure of green space. Studies mainly focused on green space around residential areas, and only one study considered participants’ activity area (considering residential, work, and travel exposure) [45]. Four studies (five articles) measured green space around a school or kindergarten [31,46,47,48,49].

Comparing different methods of green space assessment by considering studies that used more than one method (*n* = 18), there is no consistent pattern (weaker or stronger) found for a specific method in terms of the association with health outcomes. Only a few studies indicated that street view greenness [50,51] and proximity to parks [52,53] had a slightly better association with outcomes measured compared to NDVI [50,51,52,53], green space per capita [52,53], and green space coverage [50,51].

### 3.3. Mental Health Outcomes

Overall, 30 unique studies (*n* = 33 articles) comprising eight experimental (RCTs; *n* = 9 articles) and 22 observational studies measured mental health outcomes. Observational studies were 2 before–after, 1 quasi-experimental, and 19 cross-sectional (*n* = 21 articles). Sample size for RCTs ranged from 10 [35,54] to 364 [55] in each group; for BAS studies, 43 [56] and 257 [57]; and for cross-sectional and cohort studies from 150 [39] to 59, 754 [49]. RCT studies mainly focused on outcomes indicating temporary changes. Among them, mood states measured by a validated tool of the profile of mood states (PoMS; *n* = 6) [36,54,55,58,59,60] were the most common outcomes assessed. This tool comprises six domains of tension–anxiety, depression, anger–hostility, vigour, fatigue, and confusion. Other outcomes that RCT studies assessed were feelings (e.g., comfort, tension) [58,61]; relaxation and anxiety [61]; and physiological indicators of stress/relaxation such as brain activity [58,61], heart rate variability, and skin conductance [58]. The outcomes assessed by observational studies were overall mental health (*n* = 13) [16,37,39,40,44,51,62,63,64,65,66,67,68,69], depression (*n* = 3) [41,70,71], stress [51,56,72], affective state [57], attention deficit hyperactivity disorder (ADHD) [49], and children’s behavioural problems [46], all measured by subjective validated tools.

Studies with an RCT design all showed significant improvement in at least one of the mental health outcomes measured. Five of these studies were conducted among healthy university students [35,36,55,58,59,61] and two among older adults with chronic heart failure [73] or chronic obstructive pulmonary disease (COPD) [54]. Interventions were forest bathing (viewing/walking; *n* = 6) [35,36,54,59,60,61,73] for 15 min [55,59,61,73] to 1.5 h [35,60,73], viewing a façade compared to viewing a wall for a few minutes [58], walking 15 min along a roadside with various levels of trees for 7 days [55].

Similarly, observational studies all showed a positive association between green space and at least one mental health outcome, except one that did not show any association with the WHO wellbeing scale [44]. This study used a subjective method for measuring green space. The duration of follow-ups for the quasi-experimental study was four years [64] and that for the before–after studies was one day of park visiting [56,57]. Studies were conducted in a general population (*n* = 13) [37,39,44,51,62,63,64,65,66,67,69,70,74], older adults (60+ years; *n* = 5) [16,40,57,68,71], children under 18 years (*n* = 2) [46,49], and university students (*n* = 2) [56,72].

Some studies assessed the potential effects of social connection, including social cohesion, social capital (trust, reciprocity, and social group membership) [74], and social interaction (*n* = 8) [16,37,51,62,68,70,74]; physical activity [16,51,74] including walking behaviour (*n* = 5) [62]; air pollution (*n* = 4) [16,37,51,62]; stress (*n* = 3) [51,62,70]; noise (*n* = 2) [37,51]; and satisfaction with greenness [62] (*n* = 1), on the association. All studies that assessed the potential effect of social connection or physical activity found a significant impact. Two studies (66%) [51,62] found that stress mediated the effect of the association between mental health outcomes and green space [70] or street view green space [51]. Two studies (50%) found mediation effects of air pollution. One study found a mediation effect of noise (50%) [37], and another with satisfaction with greenness [70]. Some studies reported a stronger relationship in areas with a high level of urbanisation [70], low household income [74], and in people with a higher level of physical activity [64] and higher leisure satisfaction [41,67]. In a study of school children with the outcome of behavioural development, the association was stronger in boys compared to girls [37], while in another study in children, age, sex, household income, dog ownership, and residence district did not show any modification effect on the association between green space and ADHD [49].

### 3.4. General Health Outcomes

General health outcomes were measured in 16 studies. Two of the studies were cohorts with [75,76] 12 [76] to 14 years [75] of follow-ups, and the remaining studies were cross-sectional [16,39,40,41,42,43,45,75,77,78,79,80,81,82]. The sample size ranged from 150 [39] to 368, 399 [78]. Participants were older adults (60+ years) in most of the studies (*n* = 10) [16,41,75,76,77,78,79,81]. The rest of the studies included various age groups of the general population [39,42,43], adults (16–59 years) [45,82], or middle-aged [41] and older adults [79]. Self-rated health [41,42,77,78,79,82] was assessed in six studies; of those, four showed a positive association with green space [41,77,78,82]. Physical health was measured in five studies [16,39,40,43,45], and all except one [40] showed a significant association with green space. All studies that assessed frailty (*n* = 4) [75,76,81] found a negative association between disability and green space. Quality of life assessment (assessing physical and mental components) [40] and the presence of any chronic diseases [80] did not show any significant association with green space.

A few studies measured the mediating effect of physical activity [16,45] and air pollution [16]. Of those, physical activity in one study [78] partially mediated the association. A stronger protective effect of green space on disability was found among participants who were male, older (75+ years), less educated, married, had no diseases, and lived in a non-central and somewhat walkable area [81]. One study could not find any association with rural area when rural and urban samples were analysed separately. However, the sample size from the rural area was one-fourth that of the urban area [76]. In one study, leisure satisfaction [41] modified the association between the time spent in nature and a general health measure.

### 3.5. Cardiometabolic and Cerebrovascular Outcomes

There were 14 studies (20 articles) that measured cardiometabolic and cerebrovascular outcomes, consisting of seven experimental (RCTs) [35,36,60,61,73,83,84] and seven (12 articles) observational (cross-sectional) studies. Sample sizes ranged from 10 [54] to 60 [61] in each group in RCT studies and from 2410 [85] to 24,845 [86] in cross-sectional studies. Participants were adults (*n* = 6; 11 articles) [33,46,50,85,86,87,88,89,90,91,92], healthy university students (*n* = 3) [36,61,83], children (under 18 years) *n* = 1 [48], and elderly people (60+ years) [80] with coronary heart failure *n* = 3 [60,73,84]. All studies used objective measures of outcomes except for one that included measured self-reported doctor-diagnosed CVDs. Outcome measures were hypertension/blood pressure (*n* = 7) [36,48,50,60,61,83,85,86]; diabetes and glucose homeostasis markers (e.g., fasting blood glucose) (*n* = 5) [9,33,46,50,87]; dyslipidaemia and lipid profile (*n* = 3) [50,88,90]; cardiovascular disease (*n* = 2) [85,92]; stroke or cardiovascular risk measures (i.e., metabolic syndrome, risk score) (*n* = 2) [50,89]; heart rate and pulse pressure (*n* = 3) [36,60,83]; and CVD-related biomarkers including brain natriuretic peptide (BNT) and N-terminal-pro hormone BNP (NT-ProBNP); homocysteine; vaso-regulatory makers (e.g., endothelin-1, renin) [60,73,84].

The interventions were 15 min [61,83] to 1.5 h [36,60,73,84] sessions of forest walking and/or viewing for 2 [61] to 7 [60] days compared to a similar activity in an urban environment. All studies showed favourable changes in at least one outcome. All studies that measured blood pressure [36,61,83,84] and vaso-regulatory markers of endothelin-1 [35,36,60,73,84] and angiotensinogen [60,73] reported a significant beneficial effect of green space. Other vaso-regulatory markers, renin [60,84], angiotensin II, and angiotensin II type 1 and 2 receptors [60], showed significant improvements in more than 50% of studies. Pulse pressure had a decreasing trend (*n* = 2) [60] and no change was observed in heart rate [36,60,83], biomarkers of BNP and NT-ProBNP [73], and homocysteine [60].

Similarly, among cross-sectional studies, blood pressure-related outcomes [48,50,85,86] showed the most consistent negative association, with 100% of studies (*n* = 4) reporting significant results. Lipid profile or dyslipidaemia and cardio- and cerebrovascular risk scores and disease [50,85,89,92] in 100% and diabetes mellitus and glucose haemostasis in 75% of studies [33,46,87,91] were negatively and significantly associated with at least one measure of green space.

Mediation effects of physical activity (*n* = 3) [85,86,87,88,89,90,91,92], air pollution (*n* = 2) [86,88,89,90,91,92], and BMI (*n* = 2) [86,88,90,91] were measured in some studies. Of those, one study found a partial mediating effect of physical activity on hypertension, coronary heart disease, and stroke [85]; another study found mediating effects of air pollution and BMI on hypertension, diabetes mellitus, lipid profile, and metabolic syndrome [86,89,90,91,92].

Some studies found that the beneficial association is stronger [86,90] or only appears in women [48,87]. One study found a positive association between diastolic blood pressure (DBP) and green space in boys under 18 (more green space, higher DBP). Another study reported the association is stronger for participants with higher income [89] while another found a stronger association among low socioeconomic status (SES) participants [33]. For outcomes of metabolic syndrome and diabetes mellitus, the association was stronger in younger participants [89,91]. In contrast, for the outcomes of lipid profile, this was the reverse (the older, the stronger the association) [90]. One study found a stronger association among participants with lower exposure to air pollution [33].

### 3.6. Anthropometric Outcomes

Eight studies measured anthropometric outcomes [31,38,46,50,79,93,94,95] with a cross-sectional design except for one which was ecological [95]. Sample size varied from 427 [38] to 59, 540 [31], the ecological study examined 189 districts. Two of the studies were conducted in children and adolescents (<18 years) [31,46], two in middle-aged and older adults [79,93], and the rest in the general population. BMI (*n* = 4), waist circumference (*n* = 3), obesity/overweight prevalence (*n* = 5), abdominal obesity (*n* = 2), and peripheral obesity (*n* = 1) were the outcomes assessed. Six [31,50,79,93,94,95] out of eight studies showed significant inverse associations with at least one outcome of the anthropometry. Studies with null results (*n* = 2) all used subjective measures, while studies with a significant association mostly used objective measures. The assessment methods could be a reason for the inconsistency in the findings.

One study assessed outcomes for men and women separately and found a larger effect size in women for BMI compared to men [95]. Two studies [93,94] analysed the effect modification of sex, age, and education. These studies reported that the association was stronger for women, those with a lower education level, and older participants [93,94], but the association was stronger in younger participants when abdominal obesity was considered [93]. The potential impact of sex on the association was distinct in school children, with the association being stronger in boys than girls [31,46]. Older children (13+ years), city-dwellers, and those with less educated parents [93] seem to receive more benefits from green space [31].

Mediation effects of physical activity [31,79,93,94], air pollution [31,93,94], and perennial mean temperature [31] were assessed in some studies. All studies measuring air pollution [31,93,94] found that it partially mediated the association with a small effect size. Only one study found a mediation effect for physical activity [79].

### 3.7. Respiratory Diseases

A few studies (*n* = 7) measured respiratory outcomes; of them, one had an experimental design (RCT) and the rest were observational (cross-sectional *n* = 5; case–control *n* = 1) [47,52,53,80,96,97]. The sample size was 10 in each group for the RCT study [54], and for the observational studies, it ranged from 312 [53] to 75, 105 [96], except for one study that examined Shanghai’s entire population [97]. Participants were children [47,52] and middle-aged and older adults [53,54,80,96,97]. Outcomes were allergies and asthma in children [47,52], lung cancer [53,97], COPD or its markers [54,96], and the presence of any respiratory diseases [80]. The outcomes were either measured objectively [53,96,97] or subjectively with a validated questionnaire [47,52].

Asthma and wheezing in children were negatively associated with green space and [47,52] green space was neither related to eczema and rhinitis [52] nor the presence of any respiratory diseases [80]. An RCT study that assessed the effect of forest bathing on COPD biomarkers (pulmonary and activation-regulated chemokine, surfactant protein D and tissue inhibitor of metalloproteinase) showed a beneficial effect of the intervention after seven days of forest bathing [54]. However, green space was positively associated with COPD in a study [96]. This association was only significant for residents from north-eastern and northern China and was significant in the younger group (40–65 years). Lung cancer incidence was not related to residential green space in one study [53] and was negatively associated with green space when industrial park areas were included in another study [97].

One study examined the mediation effect of air pollution, doctor-diagnosed allergy symptoms, physical activity, and BMI on the potential effect of green space on asthma and allergies and found that air pollution and doctor-diagnosed allergy symptoms mediated the association [47]. Another study assessed whether sex, smoking status, or economic status has any impact on the association of COPD with green space but could not find any effect [96].

### 3.8. Mortality

Mortality was measured in five studies consisting of two cohort studies [98,99] and three ecological studies [100,101,102]. The two cohort studies had a sample of 16, 820 and 31, 618 older (65+ years) [99] and oldest-old (80+ years) [98] participants, respectively, and the ecological studies examined samples at the level of city or province in the general population. Outcomes were total mortality (*n* = 4) [98,99,100,102], respiratory mortality (*n* = 1), cardiovascular mortality (*n* = 1) [100], and cardiorespiratory mortality (*n* = 1) [101].

In all five studies, at least one outcome was significantly and inversely associated with green space. Two cohort studies found that residential NDVI at the closest time to the event was negatively associated with all-cause mortality but not the average of NDVI or the change in NDVI during the follow-up [98,99]. One study assessed factors that explain the heterogeneity in the association between air pollution (PM10) and mortality (total, respiratory, and cardiovascular) and showed that residential green space is one of the factors that modify the effect of air pollution on mortality [99]. Two other studies also indicated a significant negative association between green space and cardiorespiratory mortality [101] and age-adjusted mortality [102]. One study assessed the mediation effect of PM2.5 on mortality and found a small effect [99], and the protective effects of residential green space were more pronounced among women and those who exercised in one study [98].

### 3.9. Infectious Diseases

Four studies assessed the relationship between green space and infectious diseases [103,104,105,106]. Of those, three were ecological studies and one was a cohort study. The sample was the national population [103,105] or the population of the study area [104,106]. These studies assessed the incidence of dysentery [103,106], malaria [103,105], dengue fever [104], and tuberculosis [103], all collected from national databases. Two of the studies found an inverse association between green space and dysentery incidence [103,106]. However, other studies showed that green space is positively related to malaria [103,105], dengue fever [104], and tuberculosis [103]. One of these studies showed that the association is only positive for overall green space but not public green space [103].

### 3.10. Birth Outcomes

Three studies measured birth outcomes, including two cross-sectional studies [34,107] and one cohort study [32] with follow-up for the gestational period. The sample size varied between 4329 and 20,867 pregnant women. Outcome measures were fetal growth [32], miscarriage [34], and congenital heart failure [107], all measured objectively. All three studies showed some favourable relationships between green space and the outcomes. The cohort study showed that estimated fetal growth, head circumference, and abdominal circumference were significantly higher in the fetuses of women with more residential green space [32]. A study that assessed the modifying role of green space in the effect of maternal exposure to temperature on the risk of miscarriage found a smaller effect of temperature on the risk of miscarriage in those women living with moderately larger residential green space [34]. The last study showed that maternal residential green space had a protective effect on the development of congenital heart failure [107]. The level of residential greenness has a threshold effect at NDVI > 0.21 for congenital heart failure, and the benefit was strongest for modest values and then stabilised or gradually declined. One study observed air pollution mediated 52% of the association between green space and congenital heart failure incidence. The protective associations were stronger for urban or permanent residents, higher household income, maternal age ≤35 years, and high maternal education [107].

### 3.11. Other Health Outcomes

Serum vitamin D [108], sleep quality [109], peripheral oxygen saturation [36,83], cognitive function [110,111], markers related to immune system function such as T cell, B cell, and natural killer cell counts [35,36,54], platelet activation [35], stress marker (cortisol) and testosterone levels [35,36,54], pro-inflammatory markers such as interleukin-6 (IL-6), tumour necrosis factor-alpha (TNF-a), high-sensitivity C-reactive protein (hs-CRP), oxidative stress markers (total superoxide dismutase, malondialdehyde) (*n* = 5) [35,54,60,73,84], and the presence of various chronic diseases (e.g., joint diseases, endocrine disease) [80] were other outcomes measured.

Four of these studies were RCTs [35,36,54,60,83] that examined the effect of walking in a forest for 15 min [36,83] or 1.5 h [35,54,60] twice a day for 2 days compared to walking in an urban environment. Serum levels of cortisol, and some of the pro-inflammatory markers such as IL-6 [35,54,73], IL-8, interferon- γ, interleukin-1b [54], and the oxidative stress marker malondialdehyde [35,73] decreased after the intervention in the forest group but not in the control. Further, some markers indicating immune system function such as total B cells, cluster of differentiation 8 T cells, and natural killer T-like cells increased [35,36,54].

A cohort study reported inverse association between green space exposure for 12 years and vitamin D deficiency in a sample of 1336 older people (65+ years) reported an inverse association between green space and vitamin D deficiency. The association was stronger in men and people without disability at baseline.

Two cohort studies that used the same dataset examined if more residential green space could protect against cognitive function [110,111] and impairment [110] among older adults (65+ years) (*n* = 6995) at follow-up between 2 to 14 years later. Longitudinal analysis in one study did not show any association [110]. The cross-sectional analysis found that the participants living in the highest quartile of residential green space had lower odds of cognitive impairment than those in the lowest quartile. The second cohort indicated that this protective effect was only observed among non-Apoe epsilon 4 carriers and adults aged 65–75 years [111].

One cross-sectional study explored association between three years of residential green space exposure and sleep quality among 27, 654 participants living in a rural area. Green space was negatively and significantly associated with poor sleep quality. Physical activity and BMI did not appear to have any mediating effect. A modification effect of air pollution was observed in the green space–sleep association with a stronger association in more polluted areas. A stronger association among males and individuals with higher household income and education was also reported [109].

## 4. Discussion

This review synthesised associations between green space and health (excluding health behaviour) reported by studies conducted in mainland China. A wide range of health outcomes were reported. Overall, the review of 83 publications (73 unique studies) indicates predominantly beneficial associations between green space and an array of health outcomes, including general health, healthier BMI and anthropometry, mental health, and cardiometabolic and cerebrovascular outcomes. Of the included studies, only three examined the quality of green space, which represents a major limitation and horizon for future research. Our findings are consistent with systematic reviews that have assessed associations between green space and physical health [4] or mental health outcomes [112]. The current review found insufficient evidence to draw firm conclusions on mortality, birth outcomes, and cognitive function, and findings on respiratory and infectious outcomes were inconsistent and limited.

Publication trends indicated a rapidly growing interest in green space and health research in China, with 90% of reviewed studies published in the last four years alone (2017–2020). In addition, over half of the studies (52%) were conducted in highly urbanised areas of the country, whereas one-third were conducted nationwide. The study findings are generalisable to both sexes as most of the studies included a balanced ratio of women to men, though with differences in the ‘prevention potential’ of green spaces by gender potentially driven by factors related to quality (e.g., park safety) were under-researched. Most studies were judged to be of ‘fair’ quality with a cross-sectional design, a common limitation prohibiting minimisation of certain biases, such as self-selection and reverse causation common to studies that do not consider residential histories of study participants. Longitudinal studies and natural experiments capable of addressing biases attributable to selective (im)mobility would help to strengthen the evidence. It is worthwhile to note that, despite most studies using a cross-sectional design, almost all studies showed beneficial relationships between green space and at least one health outcome measured. Below, we further discuss findings in detail, provide suggestions for future research and then findings that are specific to particular outcomes.

This review focused on the quantity of green space and health outcomes. Among these studies, few studies considered the quality or types of green space (*n* = 3). Quality features of green space such as aesthetics, biodiversity, walkability, sport/play facilities, and safety [113] might be some of the essential factors connecting green space to health by making them attractive places to spend discretionary time [114]. For example, one of the included studies that considered the availability of evergreen trees in parks in a winter city found a negative association between these types of trees and obesity and hypertension [50]. In this instance, having information on the presence of particular vegetation types in local areas and their health impacts could inform green space generation and restoration strategies [115]. This could be especially important in areas of China where a cold climate is associated with higher CVD risk [116,117].

Evidence of effect modification via participants’ sociodemographic characteristics such as age, sex, and SES on associations between green space variables and health outcomes in the studies reviewed was inconsistent and limited. For example, some studies indicated stronger associations in women and girls [48,84,86,87,90,93,94,98], although a few had observed no difference between men and women [46,96] or stronger associations in men [109]. There is also evidence that green space might benefit people with low SES [118]. People with a low SES generally have poor health status in China and other countries [119,120]. Despite recent tremendous social and economic developments in China, socioeconomic-related health disparity has increased, favouring those with a high SES. It is suggested that focusing on lifestyle and environmental improvement can help to reduce the disparity [121]. Thus, an appropriate strategy for developing green space would be a strategy assisting with alleviating health inequality. Further studies are needed to assess the potential modification of associations between green space and health among participants with different characteristics.

Only a few studies assessed potential effect modification across levels of urbanisation [70,107] or the difference in associations between urban and rural dwellers [76,110]. While the included studies indicate that people in urban areas might obtain greater benefits from green space compared to counterparts in rural areas, this association should be studied further. This is because the green space that is typically available in rural areas can have markedly different physical characteristics and contrasting social connotations in comparison to that found in urban areas, including industrial fertiliser and pesticides, farm animals, wildlife, access issues, and potential for physical isolation due to the sparsity of human settlements [122].

Studies conducted in children and adolescents are promising but limited in outcomes such as obesity and anthropometry, mental health, and cardiometabolic risk factors such as hypertension. Green space can be important for children and adolescents’ development [23,123]. Similar to other countries, neurodevelopmental disorders in children, such as ADHD, are concerning in China [124]. In addition, considering the growing rate of childhood obesity and cardiovascular risks over the last decade in China [125,126], particularly in adolescents [127,128], research on the health benefits of green space in children and adolescents is crucial. The results of future studies in these age groups might be of interest for policymakers to target the right population for framing strategies in green space.

Most of the studies that assessed associations between green space and mental health measured overall mental health, limiting the relationship between green space and specific mental disorders such as anxiety and depression. In addition, experimental studies conducted to examine the impact of green space on mental health-related outcomes only measured the short-term changes in outcomes such as mood states or affective states. While momentary contact with green space has been shown to improve general wellbeing, sustained contact over long periods of time may be crucial to see clear impacts on more substantial mental health outcomes (e.g., psychiatric morbidity). Moreover, recent research shows that the rate of mental disorders, particularly anxiety and depressive disorders, is high in China [129]. Therefore, studies that attempt to identify evidence of potentially causal associations between green space and specific mental disorders are an important next step.

Our review showed that green space had (in 75% of studies) an inverse association with body weight and anthropometry in adults and older adults; however, evidence in younger age groups is limited. Consistent with our findings, in general, previous systematic reviews [21,130,131] reported an inverse association between obesity/overweight. Obesity, the leading risk factor for several NCDs, has become a major health concern for the Chinese healthcare system [132] and studies that evaluate the impacts of urban greening on weight status in China would be a valuable avenue for further investigation.

This review showed that green space had a negative association with hypertension/blood pressure, serum glucose abnormalities, and cardio-cerebrovascular-related biomarkers such as lipid profile. This association was particularly observed for blood pressure-related outcomes with a high consistency across studies. However, except for two studies assessing cardiovascular or cerebrovascular diseases and events [85,92], all studies focused on risk factors, limiting any assessment about the direct association of green space on cardio-cerebrovascular disease. In addition, the evidence in the elderly population is limited. Cardiometabolic and cerebrovascular diseases are some of the major causes of premature mortality and disabilities in China [2]. Future studies might consider examining associations between green space and cardiometabolic and cerebrovascular diseases. Additionally, given China’s rapidly ageing population [15], research on green space and neurodegenerative diseases would also align with emerging studies in high-income countries [133,134,135].

Findings regarding respiratory diseases are inconsistent and inadequate; two studies included in this review found a negative association between green space and asthma and wheezing and no association with eczema and rhinitis in children [47,52]. One study also found that a forest bathing trip improved pulmonary and activation-regulated chemokine, surfactant protein D, and tissue inhibitor of metalloproteinase in people with chronic obstructive pulmonary disease (COPD) [54]. While a study that examined the association between the prevalence of COPD and green space found a positive association [96], this was evident in the north-eastern and northern regions of China only [96]. Similarly, literature about the effect of green space on respiratory diseases is inconclusive, and results varied across studies [136]. This could be because the green space’s surrounding area and type of vegetation could impact the results. For example, if the green space is adjacent to sources of air pollution such as trees near traffic roads, then the exposure to such green space can be associated with exposure to pollution such as PM and nitrogen dioxide [136]. Types of vegetation and species such as pollen-producing variants planted in parks and the presence of invasive species; inappropriate garden management and maintenance activities; and the interaction of pollen with air pollution can also cause excessive pollen sources, which could cause allergic and pulmonary diseases [137,138]. Considering the high level of air pollution in China [10] and that the vegetation types are quite distinct across its different regions [139], future studies are needed considering these variations to provide direction on the effect of green space on respiratory disease and the approach to reducing its potential adverse impact and maximise green space benefits in China.

Green space (likely in conjunction with rivers and swamps) can serve as a habitat for disease vectors that increase the rate of infections [140]. There are limited studies that assessed the impact of green space on infectious disease, and results were limited (*n* = 3) and inconsistent. For example, while green space (including productive plantations and environmental protection areas) was associated with higher tuberculosis and malaria rates, no association for public green space and green space coverage ratio was found [103]. Further, an increase in green space or forest area was shown to reduce dysentery prevalence [103]. Studies need to examine infectious diseases further. It is also worth considering that SES is one of the main determinants of the prevalence of infectious diseases [103,106]. The intersection between local economic circumstances and green space conditions for occurrence of some infectious diseases may warrant further research.

Studies conducted with other outcomes such as mortality, birth outcomes, and cognitive function are limited in China. Previous reviews, in general, indicate that green space was particularly related to these outcomes. Thus, future studies might examine green spaces’ impact on these outcomes in the context of China.

About half of the studies explored the pathway by which green space may impact health outcomes. Air pollution consistently mediated the effect of green space for almost all health outcomes. Air pollution in China is still high and can contribute to the increasing rate of NCDs such as respiratory disorders [10,141]. Another pathway by which green space seemed to be effective on obesity, cardiovascular outcomes, and mental health was via physical activity. The association for physical activity was found to be partially significant in some, but not all, studies. This mediating effect was mainly seen for mental health outcomes. This could be, to some extent, due to subjective data collection methods used in some studies. The second reason could be that the health impacts of green space might have been explained more by mediating pathways other than physical activity [110]. Confirmation of this is needed with analyses using accelerometer-based physical activity measurements and robust causal mediation modelling capable of disentangling the extent that physical activity and other candidate mediators operate in parallel or serial. Other candidate mediators include, noise, temperature, social connections, satisfaction with greenness and related psychosocial factors (e.g., loneliness) [7]. However, there is inadequate evidence to affirm most of these pathways within the context of China currently.

One of the main pathways suggested to link green space to health outcomes is its influences on restorative capacities, such as attention restoration and psychophysiological stress recovery [7]. Although some of the studies in this review assessed association between green space and stress recovery, only three studies [51,62,70] considered it as a mediator. Other mechanisms suggested as pathways such as sunlight exposure, serum vitamin D levels, and development of the immune system and inflammatory responses through exposure to a range of microorganisms [142] should be examined in future studies. Sunlight [143] and serum vitamin D [144] could be pathways linking green space to psychological improvements and immune system and inflammatory developments to respiratory and chronic outcomes [142]. Among the studies included in this review, there are a few studies that measured outcomes such as serum vitamin D [108], biomarkers related to the immune system [35,36,54], and oxidative stress and inflammation [35,54,60,85], indicating a potentially beneficial effect of green space on these outcomes. However, no studies examined these variables as mediators.

### Limitations of the Review

While this review covers a wide array of research and provides valuable insight on the health impact of green space overall, there are limitations to this review that should be acknowledged. We were not able to perform a meta-analysis of the association between surrounding green space and each health outcome due to the heterogeneity of studies. First, a wide range of health outcomes was examined from study to study. Second, methods used to measure green space were inconsistent. Third, the studies’ target populations were different, particularly in terms of age groups (e.g., children, general population, or elderly) or health conditions. Fourth, some studies did not provide all data required for meta-analysis. This review also does not consider health behaviour outcomes such as physical activity, social connections, and sleep that can have meaningful impacts on NCD risk. In addition, search terms were limited to the title and abstract fields. Thus, studies that did not specify the key terms in these fields could not be retrieved. Furthermore, studies written in languages other than English were excluded from the current review; some findings may have been overlooked if published exclusively in local Chinese journals.

## 5. Conclusions

This systematic review critically synthesised evidence of associations between green space and health in China. Findings support evidence found in other countries where positive associations have been reported between green space and mental health, cardiovascular outcomes, and general health. An overall moderate level of evidence was found for the association of these outcomes considering the designs of studies, effect sizes, and consistency of results. Limited but promising findings also indicate that green space could be beneficial for birth outcomes and reducing mortality risk. However, there is a need for longitudinal studies, natural experiments, and randomised trials to determine more robust evidence of association. There is a lack of consistent evidence on the impacts of green space on respiratory and infectious diseases in China. The majority of studies have been conducted in areas with a high rate of urbanisation. Studies assessing associations between green space and health in less urbanised areas of China are limited. Air pollution, physical activity, and social connections are the top three mechanisms assessed as pathways linking green space to health outcomes. Of those, air pollution and social connection seem to be the most relevant mechanisms explaining the association, although further applications of causal mediation methods to disentangle multiple mediators operating in serial or parallel are needed. Future work on a national scale might consider cohort studies and experiments capable of testing potential mediators and effect modifiers informed by the economically, culturally, and climatically varying contexts across China.

## Figures and Tables

**Table 1:** Inclusion and exclusion criteria of the review.

Component	Included	Excluded
**Participants**	▪ Healthy or unhealthy subjects of any age or sex ▪ Human studies only	▪ Animal and lab-based studies
**Intervention/Exposure**	▪ Exposure to a high level of green space in countries in mainland China ▪ Directly measured green space, objectively or subjectively measured	▪ Exposure to green space in countries other than China ▪ Indoor greenness, sky greenness, home or private gardens ▪ Comparison between urban setting and rural or forest setting as a crude proxy for green space exposure
**Comparator**	▪ Exposure to a low level of green space in countries in mainland China	
**Outcomes**	▪ Objective and subjective physical and mental health outcomes (positive or negative)	▪ No primary or secondary health outcomes reported
**Study design**	▪ Peer-reviewed journal articles ▪ Experimental studies and trials ▪ Observational studies	▪ Conference abstracts and dissertations ▪ Reviews, qualitative studies, editorials, essays, opinion pieces ▪ Empirical studies without a health outcome
**Language**	▪ English	▪ Any languages other than English

**Table 2:** Tools used for green space exposure measurements for each health outcome category.

Tool Used *	All Studies (*n* = 73)	Health Outcomes								
		Mental Health (*n* = 30)	General Health (*n* = 16)	Cardiometabolic and Cerebrovascular (*n* = 14)	Anthropometry (*n* = 8)	Respiratory (*n* = 7)	Mortality (*n* = 5)	Infectious Diseases (*n* = 4)	Birth Outcomes (*n* = 3)	Other Outcomes (*n* = 10)
NDVI	31 (42%)	6 (20%)	6 (38%)	6 (43%)	4 (50%)	3 (43%)	2 (40%)	1 (25%)	3 (100%)	5 (50%)
SAVI	3 (4%)	1 (3)	0	3 (21%)	1 (13%)	0	0	0	0	0
Green space coverage	13 (18%)	5 (16%)	2 (13%)	1 (7%)	2 (25%)	1 (14%)	2 (40%)	3 (75%)	0	0
Proximity to green space	10 (14%)	4 (13%)	5 (31%)	1 (7%)	2 (25%)	2 (28%)	0	0	0	1 (10%)
Street view	5 (7%)	4 (13%)	0	1 (7%)	1 (13%)	0	0	0	0	0
Green space per capita	12 (16%)	2 (7%)	1 (6%)	1 (7%)	1 (13%)	3 (43%)	1 (20%)	1 (25%)	0	0
Perceived green space	4 (5%)	2 (7%)	2 (12%)	0	0	0	0	0	0	0
Other	6 (8%)	3 (10%)	3 (19%)	1 (7%)	0	0	0	0	0	1 (20%)
NA (e.g., RCT design)	12 (16%)	9 (30%)	3 (19%)	6 (43%)	0	1 (14%)	0	0	0	4 (40%)

* Some studies used more than one tool to assess green space exposure. Other tools include time spent in park, park visits, enhanced vegetation index, vegetation continuous field. NA, not applicable; NDVI, normalised difference vegetation index; RCT, randomised control trial; SAVI, soil adjusted vegetation index.

## Data Availability

No new data were created or analyzed in this study. Data sharing is not applicable to this article.

## Supplementary Materials

The following are available online at https://www.mdpi.com/article/10.3390/ijerph18189937/s1, Table S1: Search strategy; Table S2: Quality assessment of included publications; Table S3: Study summary and characteristics.
