# Supplementary material for: Green Space and Health in Mainland China: A Systematic Review
Source: Int J Environ Res Public Health. 2021 Sep 21;18(18):9937. doi: 10.3390/ijerph18189937 (PMC8472560; doi:10.3390/ijerph18189937)
Supplement: Supplementary file 1 [file ijerph-18-09937-s001.zip › ijerph-1329801-supplementary.pdf]

International Journal of
Environmental Research and Public Health

MDPI

Supplementary Materials

Table S1. Search strategy.

		**EMBASE via Ovid (1947 to 2020) – 25 November 2020**
Health outcomes	1	exp health/
	2	exp disease/
	3	(disorder* or infection* or disabilit* or symptom* or pain* or death* or injur* or illness*).ti,ab.
	4	exp Menopause/
	5	(menopaus* or perimenopaus* or peri-menopaus* or premenopaus* or pre-menopaus* or climacteric).ti,ab.
	6	(pubertal timing or puberty timing or sexual precocity or sexual prematurity or precocious puberty or premature pubarche or premature thelarche or menarche or first spermatorrhea).ti,ab.
	7	(otitis media or food-borne trematodiases).ti,ab.
	8	(maternal adj3 complication*).ti,ab.
	9	(pregnancy adj3 complica*).ti,ab.
	10	(maternal adj3 problem*).ti,ab.
	11	(obstructed labo?r or abortion or birth complication* or neonatal or birth asphyxia or birth trauma or birth sepsis or birth weight).ti,ab.
	12	((pregnancy or birth) adj outcome*).ti,ab.
	13	(congenital anomal* or neural tube defect* or congenital heart or infertile* or Miscarriage).ti,ab.
	14	(fertility or infertility).ti,ab.
	15	(cancer* or melanoma or non-Hodgkin lymphoma or leuk*mia or neoplasm*).ti,ab.
	16	(cardiomyopathy or myocarditis or atrial fibrillation or atrial flutter* or aortic aneurysm or endocarditis or cardiovascular or hypertens* or blood pressure or dyslipidemia* or Hyperlipidemia* or vascular or insulin resistan* or dyslipidaemia* or coronary or cardio* or cardiac or stroke or transient ischaemic attack* or diabet* or blood glucose).ti,ab.
	17	(life expectancy or quality of life or psychological or Mental or Well-being or emotion or Depress* or anxi* or attention or stress* or Fear* or Restorati* or ruminat* or affect* or rustrate* or agress* or lonely or loneliness or isolation or happy or happiness or resilien* or optimis* or hope* or empower*).ti,ab.
	18	(mood* or panic or dysthymic or bipolar or cyclothymic or phobia or obsessive-compulsive or somatisation or somatoform or hypochondriasis or body dysmorphic or factitious or depersonali#ation or dissociative).ti,ab.
	19	(arthritis or osteoarthritis).ti,ab.
	20	(incident* or accident* or gout or drowning or poisoning* or exposure to mechanical forces or adverse effect* of medical treatment or animal contact or health outcome* or health stat* or mortalit* or morbidit* or chronic disease* or life expectanc* or stroke* or quality?adjusted life year* or daly* or qaly* or physiological effect* or motor development* or heart rate variability or physical function or cognitive function or thyroid or metabolic or inflammat* or degenerative or ischaemic heart or "digestive system disorder" or "bone density" or impoten*).ti,ab.
	21	(autoimmun* or pneumoconiosis or respiratory or asthma or pulmonary or cirrhosis or hepati* or peptic ulcer* or gastritis or duodenitis or appendicitis or paralytic ileus or intestinal obstruction* or hernia* or pancreatitis or Alzheimer's or dementia or Parkinson's or epilepsy or multiple sclerosis or migraine* or headache* or schizophrenia or glomerulonephritis or urinary or h*moglobinopath* or an*mia*).ti,ab.
	22	(body mass index or adiposity or waist to hip or body fat or skinfold or waist circumference or body composition or overweight or over*weight or nutritional deficiency).ti,ab.
	23	exp body weight/
	24	exp obesity/
	25	exp neoplasm/
	26	sleep.ti,ab.
	27	insomnia.ti,ab.
	28	26. 1 or 2 or 3 or 4 or 5 or 6 or 7 or 8 or 9 or 10 or 11 or 12 or 13 or 14 or 15 or 16 or 17 or 18 or 19 or 20 or 21 or 22 or 23 or 24 or 25 or 26 or 27
Green space	29	(Green space* or greenspace* or green?ess or greener*).ti,ab.
	30	(green infrastructure or wilderness or wild land or natural land).ti,ab.
	31	(municipal land or community land or public land or open land or wild space or municipal space or natural space or municipal park or botanic park or park access or wild area or natural area* or green area* or woodland or shinrin-yoku or forest bathing or tree canopy).ti,ab.
	33	(urban adj3 (park* or garden* or parkland* or horticultur* or forest* or botanical or arboretum or Allotment)).ti,ab.
	34	(city park or park availability or public garden).ti,ab.
	35	(natural adj3 facilities).ti,ab.
	36	(vegetation adj3 natural).ti,ab.
	37	(belt adj3 green).ti,ab.
	38	(trial adj3 recreation).ti,ab.
	39	(Trail adj3 (green or cycl* or walk)).ti,ab.

	40	(open adj1 space).ti,ab.
	41	29 or 30 or 31 or 32 or 33 or 34 or 35 or 36 or 37 or 38 or 39 or 40
**Geographical settings**	42	exp Australia/
	43	("South East* Asia*" or "West* Pacific" or Australian*).ti,ab.
	44	exp Brunei/
	45	Bruneian*.ti,ab.
	46	exp Cambodia/
	47	Cambodian*.ti,ab.
	48	exp China/
	49	Chinese.ti,ab.
	50	Cook Islands.ti,ab.
	51	Cook Islander*.ti,ab.
	52	exp Fiji/
	53	exp Japan/
	54	Japanese.ti,ab.
	55	("Federated States" adj2 Micronesia).ti,ab.
	56	Chuukese*.ti,ab.
	57	(Kosraean* or Pohnpeian* or Yapese* or tuvalu).ti,ab.
	58	(Kiribati or Kiribatian* or Marshall Island or Marshall Islander*).ti,ab.
	59	exp Laos/
	60	Lao.ti,ab.
	61	exp Malaysia/
	62	Malaysian*.ti,ab.
	63	Malay*.ti,ab.
	64	exp Mongolia/
	65	Mongolian*.ti,ab.
	66	Nauru.ti,ab.
	67	Nauruan*.ti,ab.
	68	exp New Zealand/
	69	(New Zealander* or Niue or Niuean*).ti,ab.
	70	exp Palau/
	71	Palauan*.ti,ab.
	72	exp Papua New Guinea/
	73	Papuan*.ti,ab.
	74	exp Philippines/
	75	(Philippines or Philippine* or Filipino*).ti,ab.
	76	exp Republic of Korea/
	77	Korean*.ti,ab.
	78	exp Samoa/
	79	Samoan*.ti,ab.
	8	exp Singapore/
	81	Singaporean*.ti,ab.
	82	(Solomon Islands or Solomon Islander*).ti,ab.
	83	exp Tonga/
	84	exp Vanuatu/
	85	exp VietNam/
	86	Vietnamese.ti,ab.
	87	(Indonesian* or Sri Lankan* or Thai or Timorese or Bangladeshi* or Bhutanese or Indian or Maldivian* or Burmese or Nepali* or Fijian*).ti,ab.
	88	exp Indonesia/
	89	exp Sri Lanka/
	90	exp Thailand/
	91	exp Timor-Leste/
	92	exp Bangladesh/
	93	exp Bhutan/
	94	"Democratic People's Republic of Korea"/
	95	Korea.ti,ab.
	96	exp India/
	97	exp Maldives/
	98	exp Myanmar/
	99	exp Nepal/

	100	42 or 43 or 44 or 45 or 46 or 47 or 48 or 49 or 50 or 51 or 52 or 53 or 54 or 55 or 56 or 57 or 58 or 59 or 60 or 61 or 62 or 63 or 64 or 65 or 66 or 67 or 68 or 69 or 70 or 71 or 72 or 73 or 74 or 75 or 76 or 77 or 78 or 79 or 80 or 81 or 82 or 83 or 84 or 85 or 86 or 87 or 88 or 89 or 90 or 91 or 92 or 93 or 94 or 95 or 96 or 97 or 98 or 99
	101	28 and 41 and 100
	102	limit 101 to (human and english language)
		**MEDLINE via Ovid (1985 to 2020) – 25 November 2020**
Health outcomes	1	exp health/
	2	Disease*.ti,ab.
	3	(disorder* or infection* or disabilit* or symptom* or pain* or death* or injur* or illness*).ti,ab.
	4	exp Menopause/
	5	(menopaus* or perimenopaus* or peri-menopaus* or premenopaus* or pre-menopaus* or climacteric).ti,ab.
	6	(pubertal timing or puberty timing or sexual precocity or sexual prematurity or precocious puberty or premature pubarche or premature thelarche or menarche or first spermatorrhea).ti,ab.
	7	(otitis media or food-borne trematodiases).ti,ab.
	8	(maternal adj3 complication*).ti,ab.
	9	(pregnancy adj3 complica*).ti,ab.
	10	(maternal adj3 problem*).ti,ab.
	11	(obstructed labo?r or abortion or birth complication* or neonatal or birth asphyxia or birth trauma or birth sepsis or birth weight).ti,ab.
	12	((pregnancy or birth) adj outcome*).ti,ab.
	13	(congenital anomal* or neural tube defect* or congenital heart or infertile* or Miscarriage).ti,ab.
	14	(fertility or infertility).ti,ab.
	15	(cancer* or melanoma or non-Hodgkin lymphoma or leuk*mia or neoplasm*).ti,ab.
	16	(cardiomyopathy or myocarditis or atrial fibrillation or atrial flutter* or aortic aneurysm or endocarditis or cardiovascular or hypertens* or blood pressure or dyslipidemia* or Hyperlipidemia* or vascular or insulin resistan* or dyslipidaemia* or coronary or cardio* or cardiac or stroke or transient ischaemic attack* or diabet* or blood glucose).ti,ab.
	17	(life expectancy or quality of life or psychological or Mental or Well-being or emotion or Depress* or anxi* or attention or stress* or Fear* or Restorati* or ruminat* or affect* or rustrate* or agress* or lonely or loneliness or isolation or happy or happiness or resilien* or optimis* or hope* or empower*).ti,ab.
	18	(mood* or panic or dysthymic or bipolar or cyclothymic or phobia or obsessive-compulsive or somatisation or somatoform or hypochondriasis or body dysmorphic or factitious or depersonali#ation or dissociative).ti,ab.
	19	(arthritis or osteoarthritis).ti,ab.
	20	(incident* or accident* or gout or drowning or poisoning* or exposure to mechanical forces or adverse effect* of medical treatment or animal contact or health outcome* or health stat* or mortalit* or morbidit* or chronic disease* or life expectanc* or stroke* or quality?adjusted life year* or daly* or qaly* or physiological effect* or motor development* or heart rate variability or physical function or cognitive function or thyroid or metabolic or inflammat* or degenerative or ischaemic heart or "digestive system disorder" or "bone density" or impoten*).ti,ab.
	21	(autoimmun* or pneumoconiosis or respiratory or asthma or pulmonary or cirrhosis or hepati* or peptic ulcer* or gastritis or duodenitis or appendicitis or paralytic ileus or intestinal obstruction* or hernia* or pancreatitis or Alzheimer's or dementia or Parkinson's or epilepsy or multiple sclerosis or migraine* or headache* or schizophrenia or glomerulonephritis or urinary or h*moglobinopath* or an*mia*).ti,ab.
	22	(body mass index or adiposity or waist to hip or body fat or skinfold or waist circumference or body composition or overweight or over*weight or nutritional deficiency).ti,ab.
	23	exp body weight/
	24	exp obesity/
	25	exp neoplasm/
	26	sleep.ti,ab.
	27	insomnia.ti,ab.
	28	26. 1 or 2 or 3 or 4 or 5 or 6 or 7 or 8 or 9 or 10 or 11 or 12 or 13 or 14 or 15 or 16 or 17 or 18 or 19 or 20 or 21 or 22 or 23 or 24 or 25 or 26 or 27
Green space	29	(Green space* or greenspace* or green?ess or greener*).ti,ab.
	30	(green infrastructure or wilderness or wild land or natural land).ti,ab.
	31	(municipal land or community land or public land or open land or wild space or municipal space or natural space or municipal park or botanic park or park access or wild area or natural area* or green area* or woodland or shinrin-yoku or forest bathing or tree canopy).ti,ab.
	33	(urban adj3 (park* or garden* or parkland* or horticultur* or forest* or botanical or arboretum or Allotment)).ti,ab.
	34	(city park or park availability or public garden).ti,ab.
	35	(natural adj3 facilities).ti,ab.
	36	(vegetation adj3 natural).ti,ab.
	37	(belt adj3 green).ti,ab.
	38	(trial adj3 recreation).ti,ab.
	39	(Trail adj3 (green or cycl* or walk)).ti,ab.

	40	(open adj1 space).ti,ab.
	41	29 or 30 or 31 or 32 or 33 or 34 or 35 or 36 or 37 or 38 or 39 or 40
Geographical settings	42	exp Australia/
	43	("South East* Asia*" or "West* Pacific" or Australian*).ti,ab.
	44	exp Brunei/
	45	Bruneian*.ti,ab.
	46	exp Cambodia/
	47	Cambodian*.ti,ab.
	48	exp China/
	49	Chinese.ti,ab.
	50	Cook Islands.ti,ab.
	51	Cook Islander*.ti,ab.
	52	exp Fiji/
	53	exp Japan/
	54	Japanese.ti,ab.
	55	("Federated States" adj2 Micronesia).ti,ab.
	56	Chuukese*.ti,ab.
	57	(Kosraean* or Pohnpeian* or Yapese* or tuvalu).ti,ab.
	58	(Kiribati or Kiribatian* or Marshall Island or Marshall Islander*).ti,ab.
	59	exp Laos/
	60	Lao.ti,ab.
	61	exp Malaysia/
	62	Malaysian*.ti,ab.
	63	Malay*.ti,ab.
	64	exp Mongolia/
	65	Mongolian*.ti,ab.
	66	Nauru.ti,ab.
	67	Nauruan*.ti,ab.
	68	exp New Zealand/
	69	(New Zealander* or Niue or Niuean*).ti,ab.
	70	exp Palau/
	71	Palauan*.ti,ab.
	72	exp Papua New Guinea/
	73	Papuan*.ti,ab.
	74	exp Philippines/
	75	(Philippines or Philippine* or Filipino*).ti,ab.
	76	exp Republic of Korea/
	77	Korean*.ti,ab.
	78	exp Samoa/
	79	Samoan*.ti,ab.
	8	exp Singapore/
	81	Singaporean*.ti,ab.
	82	(Solomon Islands or Solomon Islander*).ti,ab.
	83	exp Tonga/
	84	exp Vanuatu/
	85	exp VietNam/
	86	Vietnamese.ti,ab.
	87	(Indonesian* or Sri Lankan* or Thai or Timorese or Bangladeshi* or Bhutanese or Indian or Maldivian* or Burmese or Nepali* or Fijian*).ti,ab.
	88	exp Indonesia/
	89	exp Sri Lanka/
	90	exp Thailand/
	91	exp Timor-Leste/
	92	exp Bangladesh/
	93	exp Bhutan/
	94	"Democratic People's Republic of Korea"/
	95	Korea.ti,ab.
	96	exp India/
	97	exp Maldives/
	98	exp Myanmar/
	99	exp Nepal/

	100	42 or 43 or 44 or 45 or 46 or 47 or 48 or 49 or 50 or 51 or 52 or 53 or 54 or 55 or 56 or 57 or 58 or 59 or 60 or 61 or 62 or 63 or 64 or 65 or 66 or 67 or 68 or 69 or 70 or 71 or 72 or 73 or 74 or 75 or 76 or 77 or 78 or 79 or 80 or 81 or 82 or 83 or 84 or 85 or 86 or 87 or 88 or 89 or 90 or 91 or 92 or 93 or 94 or 95 or 96 or 97 or 98 or 99
	101	28 and 41 and 100
	102	limit 101 to (human and english language)
**PsycINFO via Ovid (1806 to 2020) – 25 November 2020**		
Health outcomes	1	exp health/
	2	exp disease/
	3	(disorder* or infection* or disabilit* or symptom* or pain* or death* or injur* or illness*).ti,ab.
	4	exp Menopause/
	5	(menopaus* or perimenopaus* or peri-menopaus* or premenopaus* or pre-menopaus* or climacteric).ti,ab.
	6	(pubertal timing or puberty timing or sexual precocity or sexual prematurity or precocious puberty or premature pubarche or premature thelarche or menarche or first spermatorrhea).ti,ab.
	7	(otitis media or food-borne trematodiases).ti,ab.
	8	(maternal adj3 complication*).ti,ab.
	9	(pregnancy adj3 complica*).ti,ab.
	10	(maternal adj3 problem*).ti,ab.
	11	(obstructed labo?r or abortion or birth complication* or neonatal or birth asphyxia or birth trauma or birth sepsis or birth weight).ti,ab.
	12	((pregnancy or birth) adj outcome*).ti,ab.
	13	(congenital anomal* or neural tube defect* or congenital heart or infertile* or Miscarriage).ti,ab.
	14	(fertility or infertility).ti,ab.
	15	(cancer* or melanoma or non-Hodgkin lymphoma or leuk*mia or neoplasm*).ti,ab.
	16	(cardiomyopathy or myocarditis or atrial fibrillation or atrial flutter* or aortic aneurysm or endocarditis or cardiovascular or hypertens* or blood pressure or dyslipidemia* or Hyperlipidemia* or vascular or insulin resistan* or dyslipidaemia* or coronary or cardio* or cardiac or stroke or transient ischaemic attack* or diabet* or blood glucose).ti,ab.
	17	(life expectancy or quality of life or psychological or Mental or Well-being or emotion or Depress* or anxi* or attention or stress* or Fear* or Restorati* or ruminat* or affect* or rustrate* or agress* or lonely or loneliness or isolation or happy or happiness or resilien* or optimis* or hope* or empower*).ti,ab.
	18	(mood* or panic or dysthymic or bipolar or cyclothymic or phobia or obsessive-compulsive or somatisation or somatoform or hypochondriasis or body dysmorphic or factitious or depersonali#ation or dissociative).ti,ab.
	19	(arthritis or osteoarthritis).ti,ab.
	20	(incident* or accident* or gout or drowning or poisoning* or exposure to mechanical forces or adverse effect* of medical treatment or animal contact or health outcome* or health stat* or mortalit* or morbidit* or chronic disease* or life expectanc* or stroke* or quality?adjusted life year* or daly* or qaly* or physiological effect* or motor development* or heart rate variability or physical function or cognitive function or thyroid or metabolic or inflammat* or degenerative or ischaemic heart or "digestive system disorder" or "bone density" or impoten*).ti,ab.
	21	(autoimmun* or pneumoconiosis or respiratory or asthma or pulmonary or cirrhosis or hepati* or peptic ulcer* or gastritis or duodenitis or appendicitis or paralytic ileus or intestinal obstruction* or hernia* or pancreatitis or Alzheimer's or dementia or Parkinson's or epilepsy or multiple sclerosis or migraine* or headache* or schizophrenia or glomerulonephritis or urinary or h*moglobinopath* or an*mia*).ti,ab.
	22	(body mass index or adiposity or waist to hip or body fat or skinfold or waist circumference or body composition or overweight or over*weight or nutritional deficiency).ti,ab.
	23	exp body weight/
	24	exp obesity/
	25	exp neoplasm/
	26	sleep.ti,ab.
	27	insomnia.ti,ab.
	28	26. 1 or 2 or 3 or 4 or 5 or 6 or 7 or 8 or 9 or 10 or 11 or 12 or 13 or 14 or 15 or 16 or 17 or 18 or 19 or 20 or 21 or 22 or 23 or 24 or 25 or 26 or 27
Green space	29	(Green space* or greenspace* or green?ess or greener*).ti,ab.
	30	(green infrastructure or wilderness or wild land or natural land).ti,ab.
	31	(municipal land or community land or public land or open land or wild space or municipal space or natural space or municipal park or botanic park or park access or wild area or natural area* or green area* or woodland or shinrin-yoku or forest bathing or tree canopy).ti,ab.
	33	(urban adj3 (park* or garden* or parkland* or horticultur* or forest* or botanical or arboretum or Allotment)).ti,ab.
	34	(city park or park availability or public garden).ti,ab.
	35	(natural adj3 facilities).ti,ab.
	36	(vegetation adj3 natural).ti,ab.
	37	(belt adj3 green).ti,ab.
	38	(trial adj3 recreation).ti,ab.
	39	(Trail adj3 (green or cycl* or walk)).ti,ab.

	40	(open adj1 space).ti,ab.
	41	29 or 30 or 31 or 32 or 33 or 34 or 35 or 36 or 37 or 38 or 39 or 40
Geographical settings	42	exp Australia/
	43	("South East* Asia*" or "West* Pacific" or Australian*).ti,ab.
	44	exp Brunei/
	45	Bruneian*.ti,ab.
	46	exp Cambodia/
	47	Cambodian*.ti,ab.
	48	exp China/
	49	Chinese.ti,ab.
	50	Cook Islands.ti,ab.
	51	Cook Islander*.ti,ab.
	52	exp Fiji/
	53	exp Japan/
	54	Japanese.ti,ab.
	55	("Federated States" adj2 Micronesia).ti,ab.
	56	Chuukese*.ti,ab.
	57	(Kosraean* or Pohnpeian* or Yapese* or tuvalu).ti,ab.
	58	(Kiribati or Kiribatian* or Marshall Island or Marshall Islander*).ti,ab.
	59	exp Laos/
	60	Lao.ti,ab.
	61	exp Malaysia/
	62	Malaysian*.ti,ab.
	63	Malay*.ti,ab.
	64	exp Mongolia/
	65	Mongolian*.ti,ab.
	66	Nauru.ti,ab.
	67	Nauruan*.ti,ab.
	68	exp New Zealand/
	69	(New Zealander* or Niue or Niuean*).ti,ab.
	70	exp Palau/
	71	Palauan*.ti,ab.
	72	exp Papua New Guinea/
	73	Papuan*.ti,ab.
	74	exp Philippines/
	75	(Philippines or Philippine* or Filipino*).ti,ab.
	76	exp Republic of Korea/
	77	Korean*.ti,ab.
	78	exp Samoa/
	79	Samoan*.ti,ab.
	8	exp Singapore/
	81	Singaporean*.ti,ab.
	82	(Solomon Islands or Solomon Islander*).ti,ab.
	83	exp Tonga/
	84	exp Vanuatu/
	85	exp VietNam/
	86	Vietnamese.ti,ab.
	87	(Indonesian* or Sri Lankan* or Thai or Timorese or Bangladeshi* or Bhutanese or Indian or Maldivian* or Burmese or Nepali* or Fijian*).ti,ab.
	88	exp Indonesia/
	89	exp Sri Lanka/
	90	exp Thailand/
	91	exp Timor-Leste/
	92	exp Bangladesh/
	93	exp Bhutan/
	94	"Democratic People's Republic of Korea"/
	95	Korea.ti,ab.
	96	exp India/
	97	exp Maldives/
	98	exp Myanmar/
	99	exp Nepal/

	100	(australia or brunei or cambodia or china or fiji or japan or laos or malaysia or mongolia or "New Zealand" or "Palau" or "Papua New Guinea" or philippines or korea or samoa or singapore or tonga or tuvalu or vanuatu or vietnam or indonesia or "Sri Lanka" or thailand or "East Timor" or "Timor-Leste" or bangladesh or bhutan or india or maldives or myanmar or nepal or "Christmas Island" or khmer or kampuchea or sabah or sarawak or phillipines or phillippines or philipines or "Navigator Island*" or "Viet Nam").ti,ab.
	101	42 or 43 or 44 or 45 or 46 or 47 or 48 or 49 or 50 or 51 or 52 or 53 or 54 or 55 or 56 or 57 or 58 or 59 or 60 or 61 or 62 or 63 or 64 or 65 or 66 or 67 or 68 or 69 or 70 or 71 or 72 or 73 or 74 or 75 or 76 or 77 or 78 or 79 or 80 or 81 or 82 or 83 or 84 or 85 or 86 or 87 or 88 or 89 or 90 or 91 or 92 or 93 or 94 or 95 or 96 or 97 or 98 or 99
	101	28 and 41 and 102
	102	limit 102 to (human and english language)
		**CINALH via EBSCO – 25 November 2020**
Health outcomes	S1	(TI(disorder* or infection* or disabilit* or symptom* or pain* or death* or injur* or Illness*)) OR (AB(disorder* or infection* or disabilit* or symptom* or pain* or death* or injur* or Illness*))
	S2	MH"health+"
	S3	(MH "disease+")
	S4	MH"Menopause+"
	S5	(IT(menopaus* or perimenopaus* or "peri-menopaus*" or premenopaus* or "pre-menopaus*" or climacteric)) OR (AB(menopaus* or perimenopaus* or "peri-menopaus*" or premenopaus* or "pre-menopaus*" or climacteric))
	S6	(IT("pubertal timing" or "puberty timing" or "sexual precocity" or "sexual prematurity" or "precocious puberty" or "premature pubarche" or "premature thelarche" or "menarche" or "first spermatorrhea")) OR (AB("pubertal timing" or "puberty timing" or "sexual precocity" or "sexual prematurity" or "precocious puberty" or "premature pubarche" or "premature thelarche" or "menarche" or "first spermatorrhea"))
	S7	(IT("otitis media" or "food-borne trematodiases")) OR (AB("otitis media" or "food-borne trematodiases"))
	S8	IT(maternal n3 complication*) OR AB(maternal n3 complication*)
	S9	IT(pregnancy n3 complica*) OR AB(pregnancy n3 complica*) OR TI(maternal n3 problem*). OR AB(maternal n3 problem*)
	S10	(IT("obstructed labo#r" or abortion or "birth complication*" or neonatal or "birth asphyxia" or "birth trauma" or "birth sepsis" or "birth weight")) OR (AB("obstructed labo#r" or abortion or "birth complication*" or neonatal or "birth asphyxia" or "birth trauma" or "birth sepsis" or "birth weight"))
	S11	(TI((pregnancy or birth) n outcome*)) OR (AB((pregnancy or birth) n outcome*))
	S12	(TI("congenital anomal*" or "neural tube defect*" or "congenital heart" or infertile* or Miscarriage)) OR (AB("congenital anomal*" or "neural tube defect*" or "congenital heart" or infertile* or Miscarriage))
	S13	(TI(fertility or infertility)) OR (AB(fertility or infertility)) OR (TI(cancer* or "melanoma" or "non-Hodgkin lymphoma" or leuk*mia or neoplasm*)) OR (AB(cancer* or "melanoma" or "non-Hodgkin lymphoma" or leuk*mia or neoplasm*)) OR (TI(cardiomyopathy or myocarditis or "atrial fibrillation" or "atrial flutter*" or "aortic aneurysm" or endocarditis or cardiovascular or hypertens* or "blood pressure" or dyslipidemia* or Hyperlipidemia* or vascular or "insulin resistan*" or dyslipidaemia* o ...
	S14	(TI("life expectancy" or "quality of life" or psychological or Mental or Well-being or emotion or Depress* or anxi* or attention or stress* or Fear* or Restorati* or ruminat* or affect* or rustrate* or agress* or lonely or loneliness or isolation or happy or happiness or resilien* or optimis* or hope* or empower*)) OR (AB("life expectancy" or "quality of life" or psychological or Mental or Well-being or emotion or Depress* or anxi* or attention or stress* or Fear* or Restorati* or ruminat* or ...
	S15	TI (incident* or accident* or gout or drowning or poisoning* or "exposure to mechanical forces" or "adverse effect* of medical treatment" or "animal contact" or "health outcome*" or "health stat*" or mortalit* or morbidit* or "life expectanc*" or stroke* or "quality#adjusted life year*" or daly* or qaly* or "physiological effect*" or "motor development*" or "heart rate variability" or "physical function" or "cognitive function" or thyroid or metabolic or inflammat* or degenerative or "ischaemic ...
	S16	(TI (autoimmun* or pneumoconiosis or respiratory or asthma or pulmonary or cirrhosis or hepati* or "peptic ulcer*" or gastritis or duodenitis or appendicitis or “paralytic ileus” or "intestinal obstruction*" or hernia* or pancreatitis or Alzheimer's or dementia or Parkinson's or epilepsy or “multiple sclerosis” or migraine* or headache* or schizophrenia or glomerulonephritis or urinary or h*moglobinopath* or "an*mia*")) OR (AB (autoimmun* or pneumoconiosis or respiratory or asthma or pulmonar ...
	S17	(TI (“body mass index” or adiposity or “waist to hip” or “body fat” or skinfold or “waist circumference” or “body composition” or overweight or "over*weight" or “nutritional deficiency”)) OR (AB (“body mass index” or adiposity or “waist to hip” or “body fat” or skinfold or “waist circumference” or “body composition” or overweight or "over*weight" or “nutritional deficiency”))
	S18	MH"obesity+" OR MH"body weight+" OR MH"neoplasm+"
	S19	TI sleep* OR TI insomnia* OR AB sleep* OR AB insomnia*
	S20	(S1 OR S2 OR S3 OR S4 OR S5 OR S6 OR S7 OR S8 OR S9 OR S10 OR S11 OR S12 OR S13 OR S14 OR S15 OR S16 OR S17 OR S18 OR S19)
Green space exposure	S21	(TI(“Green space*” or greenspace* or green#ess or greener*)) OR (AB(“Green space*” or greenspace* or green#ess or greener*)) OR (TI(“green infrastructure” or wilderness or “wild land” or “natural land”)) OR (AB(“green infrastructure” or wilderness or “wild land” or “natural land”)) OR (TI(“municipal land” or “community land” or “public land” or “open land” or “wild space” or “municipal space” or “natural space” or “municipal park” or “botanic park” or “park access” or “wild area” or “na ...
	S22	TI(urban n3 park*) OR AB(urban n3 park*)

	S23	TI urban n3 parkland* OR AB urban n3 parkland* OR TI(urban w3 garden*) OR AB(urban n3 garden*) OR TI(urban n3 horticultur*) OR AB(urban n3 horticultur*) OR TI(Urban n3 forest*) OR AB(Urban n3 forest*) OR TI(Urban n3 botanical) OR AB(urban n3 botanical) OR TI(Urban n3 arboretum) OR AB(urban n3 arboretum)
	S24	TI (urban n3 Allotment) OR AB (urban n3 Allotment)
	S25	(TI (“city park” or “park availability” or “public garden”)) OR (AB (“city park” or “park availability” or “public garden”))
	S26	TI (natural n3 facilities) OR AB (natural n3 facilities) OR TI(vegetation n3 natural) OR AB(vegetation n3 natural) OR TI(belt n3 green) OR AB(belt n3 green) OR TI(trial n3 recreation) OR AB(trial n3 recreation)
	S27	TI(Trail n3 green) OR AB(trail n3 green) OR TI(trail n3 cycl*) OR AB(trail n3 cycl*) OR TI(trail n3 walk) OR AB(trail n3 walk)
	S28	TI(open n1 space) OR AB(open n1 space)
**Geographical settings**	S29	MH"Australia+" OR MH"Brunei+" OR MH"Cambodia+" OR MH"China+" OR MH"Fiji+" OR MH"japan+" OR MH"Laos+" OR MH"malaysia+" OR MH"Mongolia+" OR MH"New Zealand+" OR MH"Palau+" OR MH"Papua New Guinea+"
	S30	(MH"Philippines+" OR MH"South Korea+" OR MH"North Korea+" OR MH"Korea+" OR MH"Samoa+" OR MH"Singapore+" OR MH"Tonga+") OR MH"Vanuatu+" OR MH"VietNam+" OR MH"Indonesia+" OR MH"Sri Lanka+" OR MH"Thailand+" OR MH"East Timor+" OR MH"Bangladesh+" OR MH"Bhutan+" OR MH"India+" OR (MH"Maldives+" OR MH"Myanmar+" OR MH"Nepal+")
	S31	(TI("South East* Asia*" OR "West* Pacific" OR Australian* or Bruneian*or Cambodian* or Chinese or "Cook Islands" or "Cook Islander*" or Japanese or chuukese* or Kosraean* or Pohnpeian* or Yapese* or Kiribati or Kiribatian* or "Marshall Island" or "Marshall Islander*" or Tuvalu)) OR (AB("South East* Asia*" OR "West* Pacific" OR Australian* or Bruneian*or Cambodian* or Chinese or "Cook Islands" or "Cook Islander*" or Japanese or chuukese* or Kosraean* or Pohnpeian* or Yapese* or Kiribati ...
	S32	(TI(Indonesian* or "Sri Lankan*" or Thai or Timorese or Bangladeshi* or Bhutanese or Indian or Maldivian* or Burmese or Nepali* or Fijian*)) OR (AB(Indonesian* or "Sri Lankan*" or Thai or Timorese or Bangladeshi* or Bhutanese or Indian or Maldivian* or Burmese or Nepali* or Fijian).)
	S33	TI("Federated States” n2 Micronesia) OR AB("Federated States” n2 Micronesia)
	S34	S21 OR S21 OR S22 OR S23 OR S24 OR S25 OR S26 OR S28
	S35	S29 OR S29 OR S30 OR S31 OR S33
	S36	S19 AND S34 AND S35
		**Scopus– 25 November 2020**
**Health outcomes**	#1	((INDEXTERMS (obesity) OR INDEXTERMS ("body weight")) OR ((TITLE-ABS-KEY (disorder* OR infection* OR disabilit* OR symptom* OR pain* OR death* OR injur* OR illness*)) OR (INDEXTERMS (health)) OR (INDEXTERMS (disease)) OR (INDEXTERMS (menopause)) OR (TITLE-ABS (menopaus* OR perimenopaus* OR peri-menopaus* OR premenopaus* OR pre-menopaus* OR climacteric)) OR (TITLE-ABS ("pubertal timing" OR "puberty timing" OR "sexual precocity" OR "sexual prematurity" OR "precocious puberty" OR "premature pubarche" OR "premature thelarche" OR "menarche" OR "first spermatorrhea" OR "otitis media" OR "food-borne trematodiases")) OR (TITLE-ABS (maternal W/3 complication*)) OR (TITLE-ABS ((pregnancy W/3 complica*) OR (maternal W/3 problem*))) OR (TITLE-ABS ("obstructed labo*r" OR abortion OR "birth complication*" OR neonatal OR "birth asphyxia" OR "birth trauma" OR "birth sepsis" OR "birth weight")) OR (TITLE-ABS ((pregnancy OR birth) W/1 outcome*)) OR (TITLE-ABS ("congenital anomal*" OR "neural tube defect*" OR "congenital heart" OR infertile* OR miscarriage OR fertility OR infertility OR cancer* OR "melanoma" OR "non-Hodgkin lymphoma" OR leuk*mia OR neoplasm* OR cardiomyopathy OR myocarditis OR "atrial fibrillation" OR "atrial flutter*" OR "aortic aneurysm" OR endocarditis OR cardiovascular OR hypertens* OR "blood pressure" OR dyslipidemia* OR hyperlipidemia* OR vascular OR "insulin resistan*" OR dyslipidaemia* OR coronary OR cardio* OR cardiac OR stroke OR "transient ischaemic attack*" OR diabet* OR "blood glucose")) OR (TITLE-ABS ("life expectancy" OR "quality of life" OR psychological OR mental OR "well-being" OR emotion OR depress* OR anxi* OR attention OR stress* OR fear* OR restorati* OR ruminat* OR affect* OR rustrate* OR agress* OR lonely OR loneliness OR isolation OR happy OR happiness OR resilien* OR optimis* OR hope* OR empower* OR mood* OR panic OR dysthymic OR bipolar OR cyclothymic OR phobia OR "obsessive-compulsive" OR somatisation OR somatoform OR hypochondriasis OR "body dysmorphic" OR factitious OR depersonali?ation OR dissociative OR arthritis OR osteoarthritis)) OR (TITLE-ABS (incident* OR accident* OR gout OR drowning OR poisoning* OR "exposure to mechanical forces" OR "adverse effect* of medical treatment" OR "animal contact" OR "health outcome*" OR "health stat*" OR mortalit* OR morbidit* OR "life expectanc*" OR stroke* OR "quality adjusted life year*" OR daly* OR qaly* OR "physiological effect*" OR "motor development*" OR "heart rate variability" OR "physical function" OR "cognitive function" OR thyroid OR metabolic OR inflammat* OR degenerative OR "ischaemic heart" OR "bone density" OR impoten* OR autoimmun* OR pneumoconiosis OR respiratory OR asthma OR pulmonary OR cirrhosis OR hepati* OR "peptic ulcer*" OR gastritis OR duodenitis OR appendicitis OR "paralytic ileus" OR "intestinal obstruction*" OR hernia* OR pancreatitis OR epilepsy OR "multiple sclerosis" OR migraine* OR headache* OR schizophrenia OR glomerulonephritis OR urinary OR haemoglobinopath* OR "anaemia*" OR hemoglobinopath* OR "anemia*" OR alzheimer's OR dementia OR parkinson's)) OR (TITLE-ABS ("body mass index" OR adiposity OR "waist to hip" OR "body fat" OR skinfold OR "waist circumference" OR "body composition" OR overweight OR "over weight" OR "nutritional deficiency")))) OR (TITLE-ABS (health OR disease* OR postmenopaus* OR obesity OR obese OR "body weight")) OR (TITLE-ABS (sleep* OR insomnia*)))
**Green Space Exposure**	#2	(TITLE-ABS ("Green space*" OR greenspace* OR green$ess OR greener* OR "green infrastructure" OR wilderness OR "wild land" OR "natural land" OR "municipal land" OR "community land" OR "public land" OR "open land" OR "wild space" OR "municipal space" OR "natural space" OR "municipal park" OR "botanic park" OR "park access" OR "wild area" OR "natural area*" OR "green area*" OR woodland OR shinrin-yoku OR "forest bathing" OR "tree canopy")) OR (TITLE-ABS (urban W/3 (park* OR garden* OR parkland* OR horticultur* OR forest* OR botanical OR arboretum OR allotment))) OR (TITLE-ABS ("city park" OR "park availability" OR "public garden")) OR (TITLE-ABS ((natural W/3 facilities) OR (vegetation W/3 natural) OR (belt W/3 green) OR (trial W/3 recreation))) OR (TITLE-ABS ((trail W/3 green) OR (trail W/3 cycl*) OR (trail W/3 walk) OR (open W/1 space)))

**Geographical settings**	#3	(TITLE-ABS (australia OR brunei OR cambodia OR china OR fiji OR japan OR laos OR malaysia OR mongolia OR "New Zealand" OR "Palau" OR "Papua New Guinea" OR philippines OR korea OR samoa OR singapore OR tonga OR tuvalu OR vanuatu OR vietnam OR indonesia OR "Sri Lanka" OR thailand OR "East Timor" OR "Timor-Leste" OR bangladesh OR bhutan OR india OR maldives OR myanmar OR nepal OR "Christmas Island" OR khmer OR kampuchea OR sabah OR sarawak OR phillipines OR phillippines OR philipines OR "Navigator Island*" OR "Viet Nam")) OR (TITLE-ABS ("South east* Asia*" OR "West* Pacific")) OR ((TITLE-ABS ("Federated States" W/2 micronesia)) OR (TITLE-ABS (australian* OR bruneian* OR cambodian* OR chinese OR "Cook Islands" OR "Cook Islander*" OR japanese OR chuukese* OR kosraean* OR pohnpeian* OR yapese* OR kiribati OR kiribatian* OR "Marshall Island" OR "Marshall Islander*" OR "Lao" OR malaysian* OR malay* OR mongolian* OR nauru OR nauruan* OR "New Zealander*" OR niue OR niuean* OR palauan* OR papuan* OR philippines OR philippine* OR filipino* OR korean* OR samoan* OR si ngaporean* OR "Solomon Islands" OR "Solomon Islander*" OR indonesian* OR "Sri Lankan*" OR thai OR timorese OR bangladeshi* OR bhutanese OR indian OR maldivian* OR burmese OR nepali* OR fijian*)) OR ((I NDEXTERMS ("South Korea") OR INDEXTERMS (korea) OR INDEXTERMS (samoa) OR INDEXTERMS ("Republic of Korea") OR INDEXTERMS ("Sri Lanka") OR INDEXTERMS (vietnam) OR INDEXTERMS ("Viet Nam") OR INDEXTERMS ("East Timor") OR INDEXTERMS (timor-leste)) OR (((INDEXTERMS ("north korea")) OR (INDEXTERMS ("democratic people's republic of korea"))) OR (INDEXTERMS (australia) OR INDEXTERMS (brunei) OR INDEXTERMS (cambodia) OR INDEXTERMS (china) OR IND EXTERMS (fiji) OR INDEXTERMS (japan) OR INDEXTERMS (laos) OR INDEXTERMS (malaysia) OR INDEXTERMS (mongolia) OR IN DEXTERMS ("New Zealand")) OR (INDEXTERMS (palau) OR INDEXTERMS ("Papua New Guinea") OR INDEXTERMS (singapore) OR INDEXTERMS (tonga) OR INDEXTERMS (vanuatu) OR INDEXTERMS (indonesia) OR IN DEXTERMS (thailand) OR INDEXTERMS (bangladesh) OR INDEXTERMS (bhutan) OR INDEXTERMS (india) OR INDEXTERMS (maldi ves) OR INDEXTERMS (myanmar) OR INDEXTERMS (nepal)) OR (INDEXTERMS (philippines)))))
	#4	#1 AND #2 AND #3
	#5	#4 LIMIT-TO (LANGUAGE , "English")

Table S2. Quality assessment of included publications (n=83). For the list of questions assessed for each type of studies, see https://www.nhlbi.nih.gov/health-topics/study-quality-assessment-tools.

a. Observational cohort and cross-sectional studies

Study (Author year)	Q1	Q2	Q3	Q4	Q5	Q6	Q7	Q8	Q9	Q10	Q11	Q12	Q13	Q14	Total score	Total applicable	Ratio	Ranking (low, fair, or high)
Bao 2021 (1)	Y	Y	Y	Y	N	N	N	Y	Y	NA	Y	Y	Y	Y	10	13	0.77	High
Chang 2019 (2)	Y	Y	NR	Y	N	N	N	N	N	N	Y	N	Y	N	5	14	0.36	Low
Chen 2017 (3)	Y	Y	NA	Y	N	N	N	N	N	N	N	N	NR	Y	4	13	0.31	Low
Chen 2019 (4)	Y	Y	NR	Y	N	N	N	N	Y	N	N	Y	N	Y	6	14	0.43	Low
Chen 2020 (5)	Y	Y	NA	Y	NA	N	NA	N	Y	NA	Y	Y	NA	Y	7	9	0.78	High
Dong 2017 (6)	Y	Y	NR	Y	N	N	N	N	N	N	Y	N	Y	Y	6	14	0.43	low
Fan 2019 (7)	Y	Y	Y	Y	N	N	N	Y	Y	N	Y	NR	y	N	8	14	0.57	Fair
Fan 2020a (8)	Y	Y	Y	Y	N	N	N	Y	Y	N	Y	NR	Y	N	8	14	0.57	Fair
Fan 2020b (9)	Y	Y	Y	Y	N	Y	N	Y	Y	Y	Y	NR	Y	Y	11	14	0.79	High
Helbich 2019 (10)	Y	Y	NR	Y	N	N	N	Y	Y	N	Y	Y	Y	Y	9	14	0.64	Fair
Huang 2019 (11)	Y	Y	Y	Y	N	N	N	Y	Y	N	N	Y	Y	Y	9	14	0.64	Fair
Huang 2020a (12)	Y	Y	NR	Y	N	N	N	Y	Y	N	N	Y	NR	Y	7	14	0.50	Fair
Huang 2020b (13)	Y	Y	Y	Y	N	N	N	N	Y	Y	Y	Y	Y	Y	10	14	0.71	Fair
Huang 2020c (14)	Y	Y	Y	Y	N	N	N	N	Y	N	Y	Y	Y	Y	9	14	0.64	Fair
Hundessa 2018 (15)	Y	Y	NA	NA	N	N	N	N	Y	N	Y	Y	NA	N	5	11	0.45	Low
Ji 2019 (16)	Y	Y	NR	Y	N	Y	Y	Y	N	Y	N	Y	N	Y	9	14	0.64	Fair
Ji 2020b (17)	Y	Y	NR	Y	N	Y	Y	N	Y	y	N	Y	Y	Y	10	14	0.71	Fair
Jia 2018 (18)	Y	Y	Y	Y	N	n	n	N	Y	N		NR	Y	Y	7	14	0.50	Fair
Leng 2020 (19)	Y	Y	NA	Y	N	n	n	N	Y	N	Y	CD	N	Y	6	13	0.46	Low
Li 2019a (20)	Y	Y	Y	Y	N	n	n	Y	Y	Y	Y	Y	Y	Y	11	14	0.79	High
Li 2020 (21)	Y	Y	NA	Y	N	N	N	N	N	N	Y	Y	NA	N	5	12	0.42	Low
Liao 2019 (22)	Y	Y	NR	Y	N	N	N	N	Y	N	Y	NR	Y	Y	7	14	0.50	Fair
Liao 2020 (23)	Y	Y	NR	y	N	n	n	N	Y	n	Y	Y	NR	n	6	14	0.43	Low
Lin 2020 (24)	Y	Y	NA	Y	N	Y	y	N	Y	Y	Y	Y	Y	Y	11	13	0.85	High
Liu 2016 (25)	Y	Y	Y	Y	N	N	N	Y	N	N	Y	N	Y	Y	8	14	0.57	Fair
Liu 2017 (26)	Y	Y	NR	Y	N	N	N	N	N	N	N	N	Y	Y	5	14	0.36	Low
Liu 2019a (27)	Y	Y	Y	N	NA	NA	Y	N	Y	Y	Y	Y	Y	Y	10	12	0.83	High
Liu 2019b (28)	Y	Y	Y	Y	N	N	N	N	Y	N	Y	Y	Y	Y	9	14	0.64	Fair
Liu 2019c (29)	Y	Y	NR	Y	N	N	N	N	Y	N	Y	Y	NR	Y	7	14	0.50	Fair
Liu 2020 (30)	Y	Y	NR	Y	N	N	N	N	Y	N	Y	Y	NR	Y	7	14	0.50	Fair
Lu 2020 (31)	Y	Y	Y	Y	N	N	N	N	Y	N	N	N	Y	Y	7	14	0.50	Fair
Madaniyazi 2016 (32)	Y	Y	NA	Y	N	N	N	N	Y	N	Y	Y	NA	N	6	12	0.50	Fair
Nie 2020 (33)	Y	Y	Y	Y	Y	Y	Y	Y	N	N	Y	Y	Y	Y	12	14	0.86	High
Peng 2020 (34)	Y	Y	Y	Y	NA	N	N	Y	Y	N	Y	Y	Y	Y	10	13	0.77	High
Qiu 2019 (35)	Y	Y	y	Y	N	N	N	N	Y	N	Y	Y	Y	Y	9	14	0.64	Fair
Qu 2020 (36)	Y	Y	Y	y	N	N	N	N	Y	N	Y	Y	Y	Y	9	14	0.64	Fair
Sun 2020 (37)	Y	Y	NR	Y	N	N	N	Y	Y	y	Y	Y	Y	Y	10	14	0.71	Fair
Takano 2002 (38)	Y	Y	NA	Y	NA	N	N	N	Y	Y	Y	Y	NA	N	7	11	0.64	Fair
Tang 2020 (39)	Y	Y	NR	Y	N	N	N	N	Y	N	N	Y	NR	Y	6	14	0.43	Low
Wang 2016 (40)	Y	Y	NR	Y	N	N	N	Y	Y	N	Y	Y	Y	Y	9	14	0.64	Fair
Wang L. 2019 (41)	Y	Y	Y	Y	NA	N	N	N	Y	N	Y	Y	Y	N	8	13	0.62	Fair
Wang R. 2019a (42)	Y	Y	Y	Y	N	N	N	N	Y	N	N	Y	Y	Y	8	14	0.57	Fair
Wang R. 2019b (43)	Y	Y	Y	Y	N	N	N	Y	Y	N	Y	Y	Y	Y	10	14	0.71	Fair
Wang R. 2020 (44)	Y	Y	Y	Y	N	N	N	N	Y	N	Y	Y	Y	Y	9	14	0.64	Fair
Wang Z. 2020 (45)	Y	Y	NR	Y	N	N	N	N	N	N	Y	N	Y	Y	6	14	0.43	Low
Xiao 2020 (46)	Y	Y	Y	Y	N	N	N	Y	Y	N	Y	NR	Y	Y	9	14	0.64	Fair
Xie 2018 (47)	Y	Y	N	Y	N	N	N	N	Y	N	N	Y	Y	Y	7	14	0.50	Fair
Xie 2020 (48)	Y	Y	Y	Y	N	Y	N	N	Y	N	Y	NR	Y	Y	9	14	0.64	Fair
Xue 2019 (49)	Y	Y	NR	Y	N	Y	Y	N	N	Y	N	Y	NR	Y	8	14	0.57	Fair
Yang 2019a (50)	Y	Y	Y	Y	N	N	N	N	N	N	Y	NR	Y	Y	7	14	0.50	Fair
Yang 2019b (51)	Y	Y	Y	Y	N	N	N	Y	N	N	Y	NR	N	Y	7	14	0.50	Fair
Yang 2019c (52)	Y	Y	Y	Y	N	N	N	Y	N	N	Y	NR	N	Y	7	14	0.50	Fair
Yang 2019d (53)	Y	Y	Y	Y	N	Y	N	Y	Y	N	Y	NR	Y	Y	10	14	0.71	Fair
Yang 2020a (54)	Y	Y	N	Y	N	N	N	N	N	N	Y	N	Y	Y	6	14	0.43	Low
Yang 2020b (55)	Y	Y	Y	Y	N	N	N	N	N	N	Y	NR	N	Y	6	14	0.43	Low
Yang 2020c (56)	Y	Y	Y	Y	N	N	N	Y	N	N	Y	Y	N	Y	8	14	0.57	Fair
Yang T. 2019 (57)	Y	Y	N	Y	N	N	N	Y	Y	N	Y	Y	NR	Y	8	14	0.57	Fair
Ying 2015 (58)	Y	Y	Y	Y	N	N	N	N	Y	N	Y	Y	Y	Y	9	14	0.64	Fair

Zeng 2020a [59]	Y	Y	Y	Y	N	N	N	Y	Y	N	Y	NR	Y	Y	9	14	0.64	Fair
Zhang 2017 [60]	Y	Y	Y	Y	NA	N	N	N	Y	N	Y	Y	NA	Y	8	12	0.67	Fair
Zhang 2018 [61]	Y	Y	NR	Y	N	N	N	N	Y	N	Y	Y	NR	Y	7	14	0.5	Fair
Zhang 2019 [62]	Y	Y	NR	Y	N	N	N	N	N	N	N	N	NR	N	3	14	0.21	Low
Zhao 2019 [63]	Y	Y	NA	Y	NA	N	N	N	Y	N	N	Y	Y	N	6	12	0.50	Fair
Zhou 2017 [64]	Y	Y	NR	Y	N	N	N	N	Y	N	NR	Y	NR	N	5	14	0.36	Low
Zhou 2020 [65]	Y	Y	NR	Y	N	N	N	N	Y	N	N	Y	NR	Y	6	14	0.43	Low
Zhu 2019a-study 1[66]	Y	Y	NR	Y	N	N	N	N	Y	Y	Y	Y	Y	Y	9	14	0.64	Fair
Zhu 2019a-study 2 [66]	Y	Y	NR	Y	N	Y	Y	N	Y	Y	Y	Y	N	Y	10	14	0.71	Fair
Zhu 2019b-study 1 [67]	Y	Y	NR	Y	N	N	N	Y	Y	N	Y	Y	N	Y	8	14	0.57	Fair
Zhu 2019b-study 2 [67]	Y	Y	NR	Y	N	Y	Y	Y	Y	N	Y	Y	N	Y	10	14	0.71	Fair
Zhu 2020a [68]	Y	Y	NR	Y	N	Y	Y	Y	Y	N	Y	Y	N	Y	10	14	0.71	Fair
Zhu 2020b [69]	Y	Y	NR	Y	N	Y	Y	Y	Y	Y	Y	Y	N	Y	11	14	0.79	High
Zhu 2020c [70]	Y	Y	NR	Y	N	Y	Y	Y	Y	Y	Y	Y	N	Y	11	14	0.79	High

b. Controlled intervention studies

Study (Author year)	Q1	Q2	Q3	Q4	Q5	Q6	Q7	Q8	Q9	Q10	Q11	Q12	Q13	Q14	Total score	Total applicable	Ratio	Ranking (low, fair, or high)
Elsadek 2019 [71]	Y	NR	NR	N	NR	NA	Y	Y	Y	N	Y	NR	Y	Y	7	13	0.54	Fair
Elsadek 2019 [72]	Y	NR	NR	N	N	NA	Y	Y	Y	Y	Y	NR	Y	Y	8	13	0.62	Fair
Hassan 2018 [73]	Y	NR	NR	N	N	NA	Y	Y	Y	Y	Y	NR	Y	Y	8	13	0.62	Fair
Jia 2016 [74]	Y	NR	NR	NR	NR	Y	Y	Y	Y	Y	Y	N	Y	N	8	14	0.57	Fair
Lyu 2019a [75]	Y	NR	NR	NR	NR	Y	Y	Y	Y	Y	Y	NR	Y	Y	9	14	0.64	Fair
Lyu 2019b [76]	Y	NR	NR	NR	NR	Y	Y	Y	Y	Y	Y	NR	Y	Y	9	14	0.64	Fair
Mao 2012 [77]	Y	NR	NR	N	NR	Y	Y	Y	Y	Y	Y	N	Y	Y	9	14	0.64	Fair
Mao 2012 [78]	Y	NR	NR	N	NR	Y	Y	Y	Y	Y	Y	N	Y	Y	9	14	0.64	Fair
Mao 2017 & Wu 2017-study 1[79, 80]	Y	NR	NR	N	NR	Y	Y	Y	Y	Y	Y	N	Y	N	8	14	0.57	Fair
Mao 2017 & Wu 2017-study 2 [79, 80]	Y	NR	NR	N	NR	N	Y	Y	Y	Y	Y	N	Y	Y	8	14	0.57	Fair
Zeng 2020b [81]	Y	NR	NR	NR	NR	Y	Y	Y	Y	Y	Y	NR	Y	Y	9	14	0.64	Fair

c. Before-after studies

Study (Author year)	Q1	Q2	Q3	Q4	Q5	Q6	Q7	Q8	Q9	Q10	Q11	Q12	Total score	Total applicable	Ratio	Ranking (low, fair, or high)
Li 2019b [82]	Y	Y	N	Y	NR	Y	N	N	N	Y	N	NA	6	11	0.55	Fair
Zhou 2019 [83]	Y	Y	Y	Y	NR	Y	Y	N	Y	Y	N	NA	8	11	0.73	Fair

Int. J. Environ. Res. Public Health **2021**, 18, 9937

Table S3. Study summary and characteristics.

Author Year province	Study design Sample size (attrition)	Population (health condition, %female, age)	Environment focus	Exposure/intervention measures and follow up duration	Outcomes measured	Outcome measurement | time points measured	Results * p<0.05	Summary of findings	Mediation or effect modification
**Mental health outcomes**									
Chang 2019 [2] All	CSS 663(NR)	Urban middle-aged citizens F:50% 40- 65y	Park visit	Subjective: frequencyof leisure activity in nature (visiting park or outdoor exercise)	Depression (absence of depression)	Subjectiv e: SWBS, CES-D	Absence of depression: (+)*	Subjective frequency ofgreen space use was positively and significantly associatedwith the absence of depression.	Leisure satisfaction partially modified the association.
Dong 2017 [6] Beijing	CSS 801(89)	General populationF: 48% 48.2 ± 17.2 y	Proximity to anurban park	Baidu Map (yes/no)	Well-being	Subjective: anitem from the Warwick Edinburgh Well-being Scale	(+)*	Residential proximity tothe park was positively and significantly associated with well- being.	A significant association was found between perceived neighbourhood social environment (particularly harmonious interpersonal relationship between neighbours) and mental well-being The association between neighbourhood social environment and mental well- being is weaker in newer neighbourhoods built after 2000.

Elsadek 2019 (71) Shanghai	RCT, CO 25(0)	Healthy university students F:100% Mean 23 ± 1.5 (SE) y	IG: green façade CG: building-wall	Wall view	Alpha relative power, HRV, skin conductance<br><br>Feelings (relaxation), mood and its subcomponents (tension-anxiety, depression, anger-hostility, fatigue, confusion, vigour)	Objective: EEG, multi-sensor device<br><br>Subjective: 5-point rating scale, PoMS ǀafter the test	Alpha relative power, high frequency power, feelings (relaxation), mood (positive scales): ↑*; mood (negative subscale), low frequency/high frequency power ratio, skin conductance: ↓*(favourable)	Viewing a green façade significantly improved emotion control (alpha relative power), parasympathetic activity (high frequency power), HRV, skin conductance, relaxation, and mood.	Not measured
Elsadek 2019 (72) Shanghai	RCT, CO 364(0)	Healthy university students and visitors F: 45% Mean 23 ± 4.6 y	IG: roadside trees CG: urban road	A 15-minute walk at 4 roadsides with various levels of trees, 5 minutes washout	Mood subcomponents (tension-anxiety, depression, anger-hostility, fatigue, confusion, vigour)	Subjective: PoMs subscales, STAI, ROS, SVSǀ before and after the test	**Follow up** PoMs negative subscales, anxiety: ↓* Poms positive subscales, restoration, vitality: ↑*	Walking on the roadside with trees significantly decreased mood disturbance, anxiety. It improved the positive subscale of the mood, restoration and vitality compared to walking on the urban road.	Thermal comfort and sky view factors were strongly (rs> 0.79) correlated (inverse) with mood disorders, STAl, ROS, SVS.
Hassan 2018 (73) Sichuan	RCT, CO 60(0)	Healthy university student F: 50% 19 and 24 y, mean19.6 ± 1.4 y	IG: Forest CG: Urban environment	A 15-minute walk in a bamboo forest and a city area	EEG (high alpha and high beta relaxation), relaxation, attention	Objective: EEG Subjective: STAI-relaxation and SATI-attention SDM (comfortable, relaxed, natural) ǀ before and after the test	**Follow up** High alpha, high beta STAI relaxation, STAI attention, SDM: ↑*	Walking in a forest significantly increased relaxation, attention and comfort compared to walking in the urban environment.	Not measured
Helbich 2019 (10) Beijing	CSS 1, 250(60)	Older population living in the area for +10y F: 60% 60+ y, mean 70.7 ± 7.0 y	Residential street view green space	Images via Tencent Map at the neighbourhood level (~0.8 km buffer)	Depression	Subjective: GDS-15	1st QR=reference 2nd QR (-)* 3rd QR: (-)* 4th QR: (-)*	Street view green space was negatively and significantly associated with depression. The association was stronger in the third quartile of green space.	Not measured

Jia 2016 [74] Zhejiang	RCT, P,2-arm (1IG, 1CG) 20, n=10 at each group	COPD patients without the acute exacerbation F: NR NR y	Forest	Walking in the site for 1.5h twice a day with a 20-min rest during the walk	Mood subcomponents (tension-anxiety, depression, anger-hostility, fatigue, confusion, vigour)	Subjective: PoMs subscales ǀ before and after	null	Walking in a forest did not change the mood in COPD patients compared to walking in the urban environment.	Not measured
Li 2019b [82]	BAS 257(57)	Older population visiting a park for recreational purposes with no difficulty in walking F: 42% 60+ y, mean 69.5 ±7.5 y	Park visit	Park visit for recreational purposes measured by GPS and device attached to participants before entering to the park	Affective states (stress, depression, relaxation and contention)	Subjective: VAS ǀ before and after the visit	Relaxation and contention: ↑*; Stress, depression: ↓*	Visiting the park significantly enhanced all subcomponents of affective state compared to baseline for all participants. Participants who actively lingered in the vegetated part of the park or walked paths showed a better restoration effect than participants who were passively involved and spent less duration of park visit.	Not measured

Liao 2020 [23] Hubei	CSS 6, 039	Children F: 48% 5–6 y	Residential green space, Kindergarten Greenspace, residence–kindergarten-weighted green space	Mean NDVIs within 0.1 km radius around the residential, kindergarten or both area	Behavioural development and its subcomponents (such as social withdrawal, anxiety and depression, Immature behaviour)	Subjective: CBCL total and subscales	Residence-kindergarten-weighted NDVI: total problem development score, anxiety and depression, aggressive behaviour, social problems, somatic complaints, obsessive behaviour, split behaviour, cruel behaviour: (-)* Kindergarten NDVI: all above and immature behaviour: (-)* Residential NDVI: split behaviour, cruel behaviour: (-)* Social withdrawal, immature behaviour, delinquent behaviour: null	Kindergarten NDVI and residence-kindergarten-weighted NDVI were negatively and significantly associated with behaviour problems and their subcomponents.	Associations were stronger in boys than in girls.
Liu 2016 [25] Jiangsu	CSS 775(12)	General population F: 51% Any age	Proximity to green space	Subjective: categorical	Well-being	Subjective: WHO-5, EQLS	null	Subjective proximity to green space was not associated with well-being.	Not measured
Liu 2017 [26] Beijing	CSS 50(0)	General population F: 51.5% 10-60 y	Park visit	Subjective: time spent at the last visit to the park	Mood, relaxation	Subjective: rating scale	Mood, relaxation: null	The time spent in the last visit to the park was not associated with any change in mood and relaxation after the visit.	Not measured

Liu 2019b [28] and Wang R. 2019b [43] All	CSS 21, 06(553)	General population F: 53% 15–64 y and 64+ y who are at work, mean 44.8 ± 14.6 y	Residential green space coverage	% green space coverage within the neighbourhood by digital map	Depression	Subjective: CES-D	(−)* After adding mediators: null	Residential green space was negatively and significantly associated with depressive symptoms.	Stress, physical activity, social cohesion completely mediated the association. The relationship was strongest at the higher level of urbanisation, and higher social capital (except for security components) and weaker for higher household income.
Liu 2019c [29] Guangdong	CSS 1, 029(NR)	General population F: 50% Mean 41.2 ±13.6 y	Residential green space	Mean NDVI within a 1 km radius around the centroid of the residential neighbourhood	Well-being	Subjective: WHO-5	Direct effect: (+)* Indirect effect : (+)* Total effect: (+)*	Residential green space has positively and significantly associated with well-being measured by WHO-5.	Partial mediation (16.7% of the total effect) was found by walking behaviour, satisfaction with greenness, and social cohesion (ps < 0.01). Stress, perceived pollution had no mediation effect.
Liu 2020 [30]	CSS 1, 150(NR)	General population F: 48 Mean 39.6 ± 11.1 y	Residential green space coverage and street view green space	Green space coverage within the 1.5 km radius around the neighbourhood	Mental health	Subjective: GHQ-12	Green space: (-)* Street view green space: (-)*	Residential green space negatively and significantly has associated with mental disorders measured by GHQ-12.	Social cohesion elements of the neighbourhood attachment, community participation mediated the association but not neighbourly interaction.

Lyu 2019a [75] and Lyu 2019b [76] Sichuan	RCT, P, 4-arm (3IG, 1CG) 120(NR), n=30 at each group	Healthy university students F: 50% 19-24 y mean 21.7 ± 0.4 (SE) y	IGs: bamboo forests (YA), bamboo forests (YB), and bamboo park (DJY) CG: urban environment (CS)	Viewing the site in the morning and then walking in the afternoon each for 15 mins	Mood and its subcomponents (tension-anxiety, depression, anger-hostility, fatigue, confusion, vigour)	Subjective: PoMs丨after test	**All 3 IGs vs CG**: PoMs total score, anger-hostility, fatigue, confusion↓*; vigour ↑*<br><br>**Forest IGs (YA, YB) vs CG**: tension-anxiety, depression ↓*<br><br>**YA vs YB:** All outcomes: null<br><br>**YB and YA vs DJY**: PoMs total score and tension-anxiety, depression, fatigue↓*; vigour ↑*	All types of exposure to bamboo forest or park significantly decreased mood disturbance, anger-hostility, fatigue, confusion, and increased vigour compared to the control group. Two bamboo forests exposure significantly decreased tension-anxiety and depression but not the bamboo park. The change in mood outcomes was not different in the two bamboo forests. Forest exposure showed a higher decrease in mood disturbance and the subscales of fatigue, tension-anxiety and depression and a higher increase in vigour compared to park exposure.	Not measured
	60(NR), n=15 at each group	Sub-analysis of male participants			Mood and its subcomponents (tension-anxiety, depression, anger-hostility, fatigue, confusion, vigour)	Subjective: PoMs丨after test		tension-anxiety, depression, anger-hostility, fatigue, confusion ↓*; vigour ↑*	
Mao 2012a [77] Zhejiang	RCT, p, 2-arm 24(0), IG: 12, CG: 12	Elderly patients with essential hypertension and CHF class I-III F: NR 60-75y	IG: Forest CG: Urban environment	Walking in the site for 1.5h twice a day with a 20-min rest during the walk	Mood subcomponents (tension-anxiety, depression, anger-hostility, fatigue, confusion, vigour)	Subjective: PoMs subscales丨before and after the test	**Baseline** all outcomes: null<br><br>**Follow up** All mood subcomponents: null	No significant difference between groups at baseline and follow up.	Not measured

Mao 2012b [78] Zhejiang	RCT, P, 2-arm 20, n=10 at each group	Healthy university students F: 0% Mean 20.8±0.5 y	IG: Forest CG: Urban environment	Walking in the site for 1.5h twice a day with a 20-min rest during the walk	Mood subcomponents (tension-anxiety, depression, anger-hostility, fatigue, confusion, vigour)	Subjective: PoMs subscales ∣ before and after the test	**Baseline** all outcomes: null **Follow up** Tension-anxiety, depression, anger-hostility, fatigue: ↓*; vigour:↑*; confusion: null	No significant difference between groups at baseline. Walking in a forest significantly decreased PoMs subcomponents of tension-anxiety, depression, anger-hostility, fatigue and increased PoMs of vigour compared to walking in the urban environment.	Not measured
Mao 2017 [79] and Wu 2017 [80] Zhejiang	RCT, P 2-arm 36 (3), IG: 24 (1), CG:12 (2)	Elderly patients with CHF class I-III F: 39% 65-80, mean 71 ± 4 y	IG: Forest CG: Urban environment	Walking in the site for 1.5h twice a day with a 20-min rest during the walk	Mood subcomponents (tension-anxiety, depression, anger-hostility, fatigue, confusion, vigour	Subjective: PoMs subscales ∣ before and after the test	**Baseline** all outcomes: null **Follow up** Tension-anxiety, depression:↓*; anger-hostility, fatigue, confusion, vigour: null	No significant difference between groups at baseline. Forest bathing significantly decreased the tension-anxiety and depression subscale of mood compared to the control group.	Not measured
Qiu 2019 [35] Guangdong	CSS 1, 150(0)	General population aged 18+ y F: 48% Mean 40.6 ±11.1 y	Residential green space per capita	GIS spatial within Road Network Buffer (based on the street network and walk time), and within 0.5 km, 1 km radii around the residential area	Mental health	Subjective: GHQ-12	GIS road network buffer: (-)* GIS 0.5km: (-) GIS 1km: null	Residential green space measured within road network buffer was negatively and significantly associated with mental disorders. The association was weaker when the circular buffer measures were used.	Not measured

Tang 2020 [39] Jiangsu	CSS Total: 591 Local: 412 Laopiao: 179	Local older population (no migrants) F: 37% 60+ y Laopiao migrants: older migrants due to being dependent on children F:54% 60+ y	Proximity to park	Distance between the entrance of the surveyed community and the nearest public park.	Mental health	Subjective: one item on a 5-point Likert scale	Local: (-)* Laopiao: null	Proximity to park was positively and significantly associated with self-rated mental health in local elderly but not in elderly who migrated due to due being dependent on their children.	Social capital has a mediating effect, and when interred into the regression model, the association was no longer significant.
Wang Z. 2020 [45] Anhui	CSS 150(28)	Residents living around the Tangxi River Park within 1.5 km radius and appeared in the park F: 33% NR y	Time spent in the park (min)	Subjective: departure time minus arrival time	Mental Health	Subjective: SF-12	(+)*	Self-reported time spent in a park was positively and significantly associated with mental health.	Not measured
Wang R. 2020 [44] and Wang R. 2019a [42] Guangdong	CSS 1, 029(0)	Adult population F: 50% Mean 41.2 ±13.6 y	Residential green space, street view green space(tree), street view green space (grass)	NDVI, street view green space tree and grass within 1 km radius around the residential neighbourhood	Well-being, stress (mediator)	Subjective: WHO-5, one question on stress	**NDVI**: well-being: (+)*, stress: null<br><br>**Street view green space-Grass**: well-being (+)*, stress (-)*<br><br>**Street view green space - Tree**: well-being (+)*	Residential green space and street view green space were positively and significantly associated with well-being. Street view green space was negatively associated with stress	NDVI: Physical activity and social cohesion partially mediated the association accounting for 22% of the association, but there was no mediation effect for stress, air quality and noise. Street view greenspace: Physical activity, stress, air quality and noise, and social cohesion explained 62% of the association. Leisure satisfaction partially modified the association

Xue 2019 [49] All	Quasi-experimental 21,543(NR)	General population F: 52% 18+ y	County level green space	Mean annual NDVI at the county level	Mental health	Subjective: MHSs	OR: (+)* β: (+) per 0.05 increase in the NDVI	County level green space has been positively associated with mental health; the strength of the association varied based on the types of analysis.	Association was stronger for people with a high level of physical activity.
Yang 2019d [53] Liaoning	CSS 59,754(0)	School or kindergarten children living in the area for 2+y F: 49% Mean 10.3 ± 3.6 y	Green space around the school	NDVI and SAVI within 0.1 km, 0.5 km, 1 km radii around the school or kindergarten	ADHD	Subjective: ADHD DSM-IV questionnaire	NDVI and SAVI 0.1km, 0.5km and 1km: (-)* per 0.1 unit increase in NDVI	Green space around the school was negatively and significantly associated with ADHD.	Age, sex and household income, type of home district, and dog ownership did not show any effect modification of the association.
Yang 2020a [54] Guangdong	CSS 591(0)	Migrants to Shenzhen F: 44 18 -68 y, mean 31.4y	Perceived residential green space	Subjective: level of greenness on 5-point Likert scale	Mental health	Subjective: GHQ-12	(-)*	Perceived residential green space was negatively and significantly associated with mental disorders measured by GHQ-12.	Social cohesion and perceived air pollution and noise mediated the association.
Yang T. 2019 [57] All	CSS 11, 954	University medical students F: 56% Mean ~22	City green space per capita and coverage	Per capita public green space in a city and public green land as a proportion of the total area of a city by the National Bureau of Statistics	Uncertainty Stress, life stress	Subjective: An author-developed questionnaire	**Uncertainty Stress** Green space < 30 ha/1000 person vs 30–39 and vs 40 and over (-)*; Green space coverage < 35 ha/km2: reference vs 40 and over: (-)* **Life stress** Greenspace and green space coverage (-)	Green space per capita higher than 30 ha per 1000 persons and coverage higher than 35 ha/km2 were negatively and significantly associated with uncertainty stress. The association for life stress was weaker and not significant.	Not measured

Zhang 2018 (61) Guangdong	CSS 1, 003(NR)	General population F:51% 19-59 y	Green space coverage around individuals' activity space and proximity	Green space coverage within 1km radius around the individual's activity space (locations where an individual undertakes daily activities) and proximity to the greenspace combined as one variable	Mental health and well-being	Subjective: SF-36, WHO-5, Social Cohesion and Trust Scale, Social Well-being Scale, SSL-I	Mental health, social health: (+) *	Green space around the individuals' activity area was positively associated with mental health.	Physical activity partially mediated the association.
Zhang 2019 (62) Jiangsu	CSS 455(0)	Adult population F:50% 60+ y	Perceived residential green space	Subjective	QoL mental health subcomponents (and social relationship)	Subjective: WHOQOL-BREF	Mental health: (+)*; Social relationship: null	Perceived residential green space was positively and significantly associated with mental health subcomponents of QoL but not with overall QoL or other subcomponents.	Not measured
Zhao 2019 (63) All	CSS ~159 million	Weibo website active users (both men and women) F: NR% NR y	Residential green space coverage	% green space around the residential area from National Bureau of Statistics	Well-being and its five domains (positive emotion, engagement, relationship, meaning, accomplishment)	Subjective: Lexica analysis of social media posts based on the PERMA theory	Total well-being and its five domains: (+)*	Residential green space was positively and significantly associated with well-being and its five domains presented in social medial posts.	Not measured

Zhou 2019 (83)<br>Guizhou	BAS<br>43(NR)	Healthy university students with no major disease<br>F: 81%<br>Mean 20.74 ± SE, 0.92 y	Urban park or rural park	One day walk in the park with 4 activities of freely walk (1), play a group game (2), have lunch and do shopping (3), and enjoy the nature view (4)	Stress for university or lifer related factors	Subjective: validated stress questionnaire for Chinese students<br>before and after the test	**Urban Park**: stress for financial state, exam pressure, love-affair relationship (-)*;<br>stress for campus life, employment worry, graduate uncertainty, lesson satisfaction, teacher satisfaction, study interest, inter-communication, room-mate contact: null<br><br>**Rural park:** stress for campus life and financial state: (-)*;<br>stress for exam pressure, love-affair relationship, employment worry, graduate uncertainty, lesson satisfaction, teacher satisfaction, study interest, inter-communication, room-mate contact: null	One day walk in the urban park decreased stress scores of financial state, exam pressure and love-affair relationship compared to the baseline values and one day walk in the rural park decreased stress of campus life and financial state.<br>Other scores such as Employment worry, Graduate uncertainty, Lesson satisfaction, Teacher satisfaction, Study interest, Inter-communication, Room-mate contact did show any change after intervention.	Participants tended to perceive more decline of anxiety from social contact in the urban forest than rural.
Zhou 2020 (65)<br>Guangdong	CSS<br>972(NR)	Older population living in Guangzhou at least 6+ months<br>F: 67%<br>60+ y, mostly 60-74	Residential green space, street view green space	NDVI within 1 km radius around the district Community, digital photographs	mental health	Subjective: Self-report	NDVI: (+)*<br>Street view green space: null	NDVI was associated with better mental health.	Social integration mediated the effect of green space on mental health. Air pollution did not have a mediating effect.<br>Income, sex, residence status and marital status regulate the mediation effect.

General health outcomes

Author Year province	Study design Sample size (attrition)	Population (health condition, %female, age)	Environment focus	Exposure/intervention measures and follow ups	Outcomes measured	Method of [outcomes] measurement/ time points measured	Results * p<0.05	Summary of findings	Mediation of effect moderation

Chang 2019 [(2)] All	CSS 663(NR)	Urban middle-aged citizens F:50% 40- 65 y	Park visit	Subjective: frequency of leisure activity in nature (visiting park or outdoor exercise)	Health	Subjective: SWBS,	(+)*	Subjective frequency of green space use was positively and significantly associated with overall health.	Leisure satisfaction partially modified the association.
Chen 2017 [(3)] Sichuan	CSS Total: 6571(NR) Urban: 3402 Rural: 2439	Urban or rural residents or a combination of both F: 47% ~47 y	Residential green space	Subjective: binary question on the shortage of greens pace	Physical health	Subjective-1item	Urban and rural residents combined, urban residents: ↑* Rural resident: null	Residential green space was significantly associated with physical health in the urban residents but not in rural residents.	Not measured
Chen 2019 [(4)] Beijing	CSS 7000(3709)	General population F: 51% 16+ y, mostly 20-39 y	Proximity to park	straight-line distance in metres from each residential location to the nearest park, and within <0.5 km, 1 km, 2 km, 3 km, 4 km buffer	Self-rated health	Subjective	Proximity to park: (+)* Proximity to park <0.5km: (+)* proximity to park 1km, 2km, 3km, 4km: null	Proximity to park in <0.5km buffer was positively and significantly associated with self-rated health but not in buffers higher than 0.5km.	Not measured
Huang 2019 [(11)] Shanghai	CSS 8, 423(461)	Older population F: 49% 65+ y	Residential green space coverage, proximity to green space	% of green space within 1 km radius around the residential neighbourhood, The shortest line from a residential neighbourhood to the nearest greenspace	Self-rated health (good, fair, poor)	Subjective	% green space: (+)* Proximity to park: (+)*	Residential green space and proximity to the park were positively and significantly associated with better self-rated health.	Not measured
Huang 2020a [(12)]	CSS Total: 368, 399(NR) Urban: 96, 442 Rural: 271, 957	Older population F: 51% 60+ y	Township-level green space	NDVI within 1km radius around the centroid of their township-level division (sensitivity analysis with radii of 1.5 km, 3 km or 8 km)	Self-rated health (good, fair, poor, can't take care of oneself)	Subjective	All: (+)* Urban: (+)* Rural: (+)* per IQR increase in NDVI	Residential green space was positively and significantly associated with self-rated health in both urban and rural areas. The association was stronger in the urban area compared to the rural. *Sensitivity analyses support the study main findings.	Not measured

Liu 2017 [26] Beijing	CSS 50(0)	General population F: 51.5% 10-60 y	Park visit	Subjective: time spent at the last visit to the park	Self-rated health	Subjective	(+)*	Time spent in the last visit to the park was positively and significantly associated with an immediate change in self-report health status after the visit.	Not measured
Peng 2020 [34] All	CSS 1, 067(0)	Older population with long-term care insurance F: 59% 60+ y, mean 82.40 ±7.68 y	Residential green space	Mean NDVI within 0.25 km, 0.5 km and 1 km radii around the residential addresses	Frailty	Objective: Basic ADL, IDAL	BADL: 3rd tertile vs 1st tertile (-)* in all NDVIs IADL: Null in all NDVIs	Being in the highest tertile of residential green space protected BADL development compared to being in the lowest tertile but not for IADL.	Stronger protective effects on BADL were found among participants who were male, older (≥75 years), had lower education, were married, had no diseases, and lived in a non-central and somewhat walkable area. The association for IADL was significant in participants with lower education and who lived in a non-central and somewhat walkable area.
Wang Z. 2020 [45] Anhui	CSS 150(28)	Residents living around the Tangxi River Park within 1.5 km radius and appeared in the park F: 33% NR y	Time spent in the park (min)	Subjective: departure time minus arrival time	Physical Health	Subjective: SF-12	(+)*	Self-reported time spent in a park was positively and significantly associated with physical health.	Not measured
Xie 2018 [47] Hubei	CSS 731(31)	Older population appeared in public space spaces F: 57% 60+ y	Accessibility to park	Nearest distance from the residence to the nearest parks based on the road network	Overall chronic health conditions	subjective	null	Accessibility to the park did not show any association with subjective chronic health conditions.	Not measured

Ying 2015 (58) Shanghai	CSS 1, 100(0)	Adults independently ambulatory with intact cognition F: NR 46-80 y	Residential green space per capita, proximity to park	Green space coverage within 0.5 km radius of residential area per capita, from a national database	Self-rated health	Subjective	Green space/person: (+)* Proximity to park: null	Residential green space was significantly associated with self-rated health. No association was found for proximity to the park.	Not measured
Zhang 2018 (61) Guangdong	CSS 1, 003	General population F: 51% 19-59 y	Green space coverage around individual's activity space and proximity	Green space coverage within 1km radius around the individual's activity space (locations where an individual undertakes daily activities) and proximity to the greenspace combined as one variable	Physical health	Subjective: SF-36	Total effect: (+)* Direct effect: null	Green space around individuals' activity areas was positively associated with physical health.	Physical activity fully mediated the association between green space and physical health.
Zhang 2019 (62) Jiangsu	CSS 455(0)	Adult population F:50% 60+ y	Perceived residential green space	Subjective	QoL and physical health subcomponent	Subjective: WHOQOL-BREF	QoL total, physical health: null	Perceived residential green space was not associated with the overall QoL or physical health.	Not measured
Zhou 2020 (65) Guangdong	CSS 972(NR)	Older population living in Guangzhou for 6+ months F: 67% 60+ y, mostly 60-74 y	Residential green space, street view green space	NDVI within 1 km radius around the district Community, digital photographs	Physical health	Subjective: Self-report	NDVI: (+) Street view green space: (+)*	NDVI was associated with better physical health (ns). Street view green space was significantly associated with better physical health	Physical activity mediated the effect of the streetscape on physical health. No mediating effect for air pollution was found.
Zhu 2019a-study 2 (66) All	CS, pros 19,076 for ADL outcome 15,656 for IADL outcome	Older population from CLHLS cohort F: 59% 65+ y	Residential green space	Mean NDVI within 0.5 km radius around the residential address Follow up: 14 y	Frailty	Subjective: ADL, IADL	1st QR=ref 2nd and 3rd QR: ADL, IADL: null 4th QR: ADL, IADL (-)* 0.1-unit of NDVI: ADL, IADL: (-)* Developing disabilities among healthy participants: (-)*	Baseline green space was negatively related to disabilities in follow-ups and developing disabilities in healthy participants.	Not measured

Zhu 2019a-study 1 [66] All	CSS 38,679(1, 876) for ADL outcome 33,797 (1, 481) for IADL outcome	Older population from CLHLS cohort F: 59% 65+ y, mean 88±11.5 y	Residential green space	Mean NDVI within 0.5 km radius around the residential address	Frailty	Subjective: ADL, IADL	1st QR=reference 2nd to 4th QR: ADL, IADL: (-)* 0.1-unit of NDVI: ADL, IADL: (-)*	Participants living in the higher quartile of residential greenness had lower ADL and IADL disabilities.	not measured
Zhu 2020b [69] All	CS, pros Total: 34,342 (18, 104) Urban: 3, 266 Rural: 12, 972	Older population (rural and urban) from CLHLS cohort F: 56% 65+ y, mean 83.0 ± 11.5 y	Residential green space	Baseline mean NDVI within 0.5 km radius around the residential addresses Follow up: 12 y	Frailty development, frailty change over time	Subjective: FI	**Total:** 4th QR vs 1st QR: FI (-)*, 0.1-unit of NDVI: FI and FI change (-)*<br><br>**Urban:** 2nd QR vs 1st QR (-)*, 0.1-unit of NDVI (-)*<br><br>**Rural**: null	Residential green space is significantly protected from frailty development and changes when analysed for all participants and urban residents but not for rural residents.	Not measured

Cardiometabolic and cerebrovascular

Author Year province	Study design Sample size (attrition)	Population (health condition, %female, age)	Environment focus	Exposure/intervention measures and follow ups	Outcomes measured	Method of [outcomes] measurement/ time points measured	Results * p<0.05	Summary of findings	Mediation of effect moderation
Fan 2019 [7] and Fan 2020a [8] Xinjiang	CSS 4, 772(37)	Healthy Uyghur adults living in the area at for +2 y F: 65% 47.5 ± 14.2 y	Residential green space	NDVI and SAVI within 0.1 km, 0.3 km, 0.5 km, and 1 km radii around the residential address	Lipid profile (LDL, HDL, TC, TG, hyperlipidaemia, hypercholesterolemia, hypoalphalipoproteinemia, hypertriglyceridemia), fasting glucose, diabetes mellitus	Objective: blood test	**NDVI and SAVI 0.1km**: hypoalphalipoproteinemia (-)*; rest of outcomes: null<br><br>**NDVI and SAVI 0.3km**: TG(-)*; rest of outcomes: null<br><br>**NDVI and SAVI 0.5km**: all outcomes null<br><br>**NDVI and SAVI 1km**: diabetes mellitus: (-)*; rest of outcomes: null	Residential green space was negatively and significantly associated with hypoalphalipoproteinemia and TG levels at 0.1 km and 0.3 km buffers, respectively but not other buffers of 0.5km and 1km. Green space was negatively associated with diabetes mellitus at the buffer of 1km only.	Associations of greenness with blood lipids were independent of physical activity, BMI, and air pollution. The protective effects of greenness on diabetes prevalence were significant only in women. However, none of the interactions was statistically significant.

Hassan 2018 (73) Sichuan	RCT, CO 60(0)	Healthy university student F: 50% 19-24 y, mean19.6 ± 1.4 y	IG: Forest CG: Urban environment	A 15-minute walk in a bamboo forest and a city area for 2 days (24 hr wash out)	SBP, DBP	Objective before and after	SBP, DBP: ↓*	Forest walking significantly decreased SBD and DBP compared to an urban walk among health university students.	Not measured
Jia 2018 (18) Anhui	CSS Total: 2, 410 (466) Middle aged: 860 Older age: 1, 084	Middle aged or older population F: 58% Middle aged: 40-59 y Older aged: 60+ y	Residential green space	Neighbourhood NDVI	Hypertension, coronary heart disease, stroke	Objective	All outcomes any age group: (-)*	Residential green space was negatively associated with hypertension, coronary heart disease and stroke in both age groups.	42.9% (16.7%, 104.8%) of associations was mediated by physical activity.
Leng 2020 (19) Heilongjiang	CSS 5, 342(1187)	Adult population F: 48% Mean 54.6 ±10.3 y	Residential green space coverage, green view index	The proportion of green space area within neighbourhood divided by the total land area of the neighbourhood-using database, images of the site	Hypertension, dyslipidaemia, diabetes mellitus, cardiovascular health score, stroke risk	Objective	**% green space**: hypertension, stroke risk (-)*; cardiovascular health score (+)* **Green view index**: dyslipidaemia, hypertension, stroke risk (-)*; cardiovascular health score (+)* **% green space and Green view index:** diabetes mellitus: null	Residential green space coverage and green view index were negatively and significantly were associated with cardiovascular risk factors except for diabetes mellitus. Dyslipidaemia was negatively associated with the green view index only.	Not measured
Liao 2019 (23) Hubei	CSS 6883(76)	pregnant women attended first parental visit F: 100% Any age	Residential green space	NDV within a 300-m circular buffer area around the residential address (sensitivity analysis with 1km radius)	Maternal fasting glucose, 1-h glucose levels, 2-h glucose levels, gestational glucose intolerance and diabetes mellitus	Objective	All outcomes: (-)* per 1-SD green space increase with a dose-response relationship	Residential green space was negatively and significantly associated with maternal blood glucose levels, impaired glucose tolerance, and gestational diabetes mellitus.	Not measured

Mao 2012 [77] Zhejiang	RCT, P, 2-arm 24(0), IG: 12, CG: 12	Elderly patients with hypertension and CHF class I-III F: NR 60-75 y	IG: Forest CG: Urban environment	Walking in the site for 1.5h twice a day with a 20-min rest during the walk for 7 days	ET-1, AT1, AT2, renin, angiotensinogen, Ang II, homocysteine SBP, DBP, heart rate, pulse pressure	Objective: blood test before and after the test	**Baseline** all outcomes: null **Follow up** ET-1, AT1, angiotensinogen, SBP, DBP (↓)* AT2, PP: ↓(ns) renin, Ang II, homocysteine, heart rate: null	No significant difference between groups at baseline. Walking in forest decreased cardiovascular disease-related markers ET-1, AT1, angiotensinogen ($p<0.05$), AT2 (ns) and blood pressures ($p<0.05$) and pulse pressure (ns) compared to walking in the urban environment.	Not measured
Mao 2012 [78] Zhejiang	RCT, P, 2-arm 20, n=10 at each group	Healthy university students F: 0% Mean 20.8±0.5 y	IG: Forest CG: Urban environment	Walking in the site for 1.5h twice a day with a 20-min rest during the walk for 2 days	ET-1,	Objective: blood test before and after the test	**Baseline**: null **Follow up**: ↓*	No significant difference between groups at baseline. Walking in a forest significantly decreased ET-1.	Not measured
Mao 2017 [79] and Wu 2017 [80] Zhejiang	RCT, P 2-arm 36 (3), IG: 24 (1), CG:12(2)	Elderly patients with CHF class I-III F: 39% 65-80 y, mean 71 ± 4 y	IG: Forest CG: Urban environment	Walking in the site for 1.5h twice a day with a 20-min rest during the walk for 4 days	BNP, NT-ProBNP, ET-1, AT1, AT2, renin, angiotensinogen, Ang II	Objective: blood test before and after the test	**Baseline** all outcomes: null **Follow up** BNP, ET-1, renin, angiotensinogen, Ang II :↓*; AT2: ↑*(compared to baseline); AT1, NT-ProBNP: null	No significant difference between groups at baseline. Forest bathing significantly decreased cardiovascular disease-related markers (BNP, ProBNP, ET-1, renin, angiotensinogen, Ang II) compared to walking in the urban environment (CG).	Not measured

Qu 2020 (36) Guangdong	CSS 5, 237(0)	Pregnant women without congenital malformations F: 100% Mean 28.0 ± 4.7 y	Residential green space	NDVI within 0.25 km, 0.5 km, 1 km, 3 km radii around the residential address during pregnancy	Gestational diabetes mellitus	Objective	NDVI 0.25km, 0.5km, 1km: (-)* NDVI 3km: null	Residential green space was negatively and significantly was associated with gestational diabetes mellitus at 0.25km, 0.5km, 1km buffers but not at 3km buffer.	The protective effect of greenness on gestational diabetes mellitus was stronger among participants with lower socioeconomic status and in environments with lower-level air pollutants.
Wu 2017-study 2 Zhejiang	RCT, P, 2-arm 23(0), IG: 12, CG: 11	Elderly patients with CHF class I-III F:46% 65-80 y, mean 72.9 ± 5.9 y	IG: Forest CG: Urban environment	Walking in the site for 1.5h twice a day with a 20-min rest during the walk for 4 days	ET-1, renin	Objective: blood test before and after the test	**Baseline** ET-1: null, renin: ↓* in CG **Follow up**: ET-1, renin: ↓*	Renin concentration was lower in the intervention group at baseline. Walking in the forest significantly decreased ET-1 and renin compared to walking in the urban environment.	Not measured
Xiao 2020 (46) Liaoning	CSS 9, 354(0)	School children living in the area for 2+y F: 49% 4-18y, mean 10.9 ± 2.6 y	Green space around the school	NDVI and SAVI within 0.5 km radius around each school (sensitivity analysis with radii of 0.1 km, 1 km)	SBP, DBP, hypertension	Objective	NDVI and SAVI: SBP, hypertension: (-)*; DBP: null per 0.1 unit increase in green space	Green space around the school was negatively and significantly associated with SBP and hypertension prevalence but not DBP.	The association for boys was positive (the higher greenspace, the higher DBP, while in girls was reverse.
Xie 2018 (47) Hubei	CSS 731(31)	Older population appeared in public space spaces F: 57% 60+ y	Accessibility to park	Nearest distance from the residence to the nearest parks based on the road network	cardio-cerebral vascular diseases	Subjective	1st QR=reference 2nd QR: null 3rd QR: null 4th QR: (-)*	Accessibility to park was associated with a lower odds of cardio-cerebral vascular diseases at quartile 4 compared to the quartile of 1.	Not measured

Yang 2019a [(50)], Yang 2019c [(52)], Yang 2020b [(55)], Yang 2020c [(56)] Liaoning	CSS 24, 845(0), 15, 477 outcomes with blood test	Healthy urban dwellers with 5+ y residence in the study community F ~46% 18-74 y	Residential green space	NDVI, SAVI (and VCF for some outcomes) within 0.5 km (and 1km for sensitivity analysis) around each health community centroid	Metabolic syndrome, SBP, DBP, hypertension prevalence, diabetes mellitus, fasting glucose, 2-h glucose, fasting insulin, 2-h insulin, HOMA-IR, HOMA-B Lipid profile (TC, TG, HDL-C), hypercholesterolemia, hypertriglyceridemia, hypoalphalipoproteinemi, Lifetime CVD status (such as myocardial infarction, cerebral haemorrhage)	Objective: blood test and based on Chinese guidelines (sensitivity analysis with various US, international and local guidelines)	NDVI and SAVI (and VCF) .05km: metabolic syndrome, SBP, DBP, hypertension prevalence, diabetes mellitus, 2-h glucose, fasting insulin, 2-h insulin, HOMA-IR, HOMA-B, TC, TG, hypercholesterolemia, hypertriglyceridemia, lifetime CVD status: (-)*; HDL-C: (+)*; fasting insulin, hypoalphalipoproteinemia: null per IQR increase in green space	Residential green space measured by NDVI, SAVI (and VCF) was negatively and significantly associated with metabolic syndrome, hypertension, SBP, DBP, diabetes mellitus and its blood markers except for fasting insulin, hypercholesterolemia, hypertriglyceridemia, blood TC, TG, LDL-C levels and CVD events. It was also positively associated with blood HDL-C levels. Sensitivity analysis supported the main findings.	Air pollution and BMI explained 2.1–78% of the associations for most of the outcomes. No mediating effect for physical activity. Greater association for the metabolic syndrome was shown in participants with higher household income and younger age. Association for blood pressure outcomes was stronger among women than men. Association for diabetes mellitus prevalence was stronger in younger participants. Associations for lipid profile outcomes were stronger for women and older individuals.
Zeng 2020b [(81)] and Lyu 2019b [(76)] Sichuan	RCT, P, 4-arm (3IG, 1CG) 120(NR), n=30 at each group	Healthy university students F:50% 19-24 y, mean 21.7 ± 0.4 (SE) y	IGs: bamboo forests (YA), bamboo forests (YB), and bamboo park (DJY) CG: urban environment (CS)	3 days of viewing the site in the morning and then walking in the afternoon each for 15 mins	SBP, DBP, heart rate	Objective | before and after the test	**IG1 vs CG**: SBP, DBP after walking and viewing: ↓ **IG2 vs CG**: SBP, DBP, heart rate after walking and viewing ↓ **IG3 vs CG**: SBP after walking ↓; heart rate and DBP null	Viewing a bamboo forest decreased SBP, DBP, heart rate decreased (ns) after walking and viewing the bamboo forests. SBP decreased after walking (ns) in the bamboo park compared to walking or visiting an urban environment.	Not measured

	60(NR), n=15 at each group	Sub-analysis of male participants					SBP ↓*; DBP, HR: null	All types of exposure to the bamboo forests or a park significantly decreased SBP compared to the control group.	
**Anthropometric outcomes**									
**Author Year province**	**Study design Sample size (attrition)**	**Population (health condition, %female, age)**	**Environment focus**	**Exposure/intervention measures and follow ups**	**Outcomes measured**	**Method of [outcomes] measurement/ time points measured**	**Results * $p<0.05$**	**Summary of findings**	**Mediation of effect moderation**
Bao 2021 [1] 7 provinces	CSS 59, 540(219)	Children and adolescents F: 51.3% Median 11 (IQR 9-15) y	Green space around the school	NDVI and SAVI within 0.1 km, 0.5 km, and 1 km radius around the school	Overweight, obesity, Z-score BMI, WC	Objective	All outcomes NDVI0.1km: (-)* NDVI0.5km: (-)* NDVI1km:(-) * per IQR increase in NDVI	Green space around the school was negatively and significantly associated with obesity/overweight and anthropometry.	PM2.5 partially mediated the association but not Nitrogen dioxide and physical activity The association for the obesity/overweight outcome was stronger among older children (age≥13 years), boys, city-dwellers and those with lower educated parents.

Huang 2020b [13] All	CSS 13, 367(1, 255)	Middle-aged population<br>F: 53%<br>50+ y	Residential green space	NDVI within 1 km radius around the residential area (sensitivity analysis with radii of 0.5 km, 1.5 km, 2 km, and 3 km)	Overweight/obesity, abdominal obesity	Objective	Overweight/obesity: (-)*<br>Abdominal obesity: (-)*	Residential green space was negatively and significantly associated with overweight/obesity. Sensitivity analyses support the study main findings.	PM2.5 suppressed the association. Physical activity and the perennial mean temperature had not mediated effect. For obesity outcome, the association was stronger in women and older participants, and for abdominal obesity, the association was stronger in female, younger participants and lower educated participants.
Huang 2020c [14] Liaoning	CSS<br>24, 845(0)	Healthy urban dwellers with 5+ y residence in the study community<br>F: 49%<br>18-74 y	Residential green space	Mean NDVI (and SAVI as sensitivity analysis) in 0.5 km and 1 km radii around each study health community's centroid	BMI, WC, peripheral obesity, abdominal obesity	Objective	All outcomes except for WC<br>NDVI0.5-km: (-)*<br>NDVI1-km: (-)* per IQR increase in NDVI	Residential green space was negatively and significantly associated with BMI and peripheral and central obesity. Sensitivity analyses support the main findings.	Air pollution partially (7.2- 11.1%) mediated the association but not physical activity. Association was stronger for women, older participants, and those with lower household incomes.
Leng 2020 [19] Heilongjiang	CSS<br>5, 342(1187)	Adult population<br>F: 48%<br>Mean 54.6 ±10.3 y	Residential green space coverage, green view index	The proportion of green space area within neighbourhood divided by the total land area of the neighbourhood-using database, images of the site being analysed via visual interpretation software	Overweight/obesity,	Objective	% green space: (-)*<br>Green view index: (-)*	Residential green space coverage and green view index negatively and significantly were associated with obesity and overweight.	Not measured

Liao 2020 [23] Hubei	CSS 6, 039	Children F:48% 5–6 y	Residential green space, kindergarten green space, residence–kindergarten-weighted green space	Mean NDVIs within 0.1 km radius around the residential, kindergarten or both area	Obesity	Subjective: CBCL subscale	null	Kindergarten NDVI residence NDVI did not show any association with self-report obesity.	NA
Lu 2020 [30] Shanghai	CSS 427(24)	General population who moved to a new green city F: ~54 18-80 y	Proximity to green space	Subjective: time to nearest green space	BMI	Subjective	null	Subjectively measured proximity to the park has no association with self-reported BMI.	Not measured
Ying 2015 [58] Shanghai	CSS 1, 100(0)	Adults independently ambulatory with intact cognition F: NR 46-80 y	Residential green space per capita, proximity to park	Green space coverage within 0.5 km radius of residential area per capita, from the national database	BMI, Overweight/obesity	Subjective Objective	**Green space/ person:** BMI (-)*, overweight/overweight: null<br>**proximity to park:** BMI, overweight/ obesity: (-)*	Residential green space and proximity to the park were negatively associated with BMI. Proximity to the park was also associated with overweight/ obesity prevalence.	Physical activity mediated the association
Zhou 2017 [64] All	ES 189 districts	General population F: NR 35–49 y	District level green space coverage	% green space using Digital Land Use Map	BMI (man), BMI (woman), obesity (man), obesity (woman), obesity (total):	NR	BMI(man) , BMI (woman), obesity (man), obesity (woman), obesity (total): (-)*	District level green space coverage was negatively and significantly associated with BMI and obesity in both men and women.	not measured

Respiratory outcomes

Author Year province	Study design Sample size (attrition)	Population (health condition, %female, age)	Environment focus	Exposure/intervention measures and follow ups	Outcomes measured	Method of [outcomes] measurement/ time points measured	Results * p<0.05	Summary of findings	Mediation of effect moderation

Fan 2020b [9] All	CSS Total: 75, 107 (8355) COPD: 9, 134	Adults lived in the area for the last +6 months, no individuals stayed in a communal residence F: non-COPD: 54% COPD: 37% 40+ y, mean non-COPD: 53.3 ± 14.3 y, COPD: 61.6 ± 13.6 y	Residential green space	Mean of 5-year NDVI preceding the survey within 0.1 km, 0.3 km, 0.5 km, 1 km, 2 km and 3 km radii around the residential community	COPD	Objective: Spirometry	(+)* per IQR increase in NDVI	Higher levels of residential green space was associated with an increased risk of COPD across all NDVI buffers.	The association was significant in residents from Northeastern and Northern China, while for other areas, the associations were null. The association was significant in the younger age group but not, the older aged. No significant differences between males and females among different smoking statuses or economic development.
Jia 2016 [74] Zhejiang	RCT, P, 2-arm (1IG, 1CG) 20, n-10 at each group	COPD patients without a current exacerbation F: NR NR y	IG: Forest CG: urban environment	Walking in the site for 1.5h twice a day with a 20-min rest during the walk for 7 days	Pulmonary and activation-regulated chemokine, surfactant protein D, tissue inhibitor of metalloproteinase	Objective: blood test before and after	All pulmonary markers: ↓*	Forest walking reduced biomarkers related to COPD compared to walking in the urban environment.	Not measured
Li 2019a [20] Jiangsu	CSS 5, 891(248)	School children F: 48.2 Mostly 12-15 y	Residential green space, proximity to park	NDVI within a 0.1 km, 0.2 km, 0.5 km and 1km radius around the residential area, distance from residential address to the nearest park using GIS	Current asthma, ever eczema, ever rhinitis	Subjective: doctor-diagnosis and ISAAC	**NDVIs:** all outcomes: null per IQR increase in NDVI<br><br>**Proximity to park**: current asthma, ever asthma 3rd QR(-) and 4th QR (-)*	There was no association between residential greenness and respiratory and allergic outcomes found. Proximity to park negatively associated with current asthma and ever asthma at 3rd (>903m, ns) and 4th quartile (>1348 m, $P<0.05$).	Not measured

Int. J. Environ. Res. Public Health **2021**, 18, 9937

Wang 2016 (40) Shanghai	CCS Case:156 Control:156	Case: Lung cancer patients living in the area for 5+ y Control: Pair patients visited hospital living in the area for 5+ y F: 62% Mean ~61±12.3 y	Residential Green space per capita, proximity to park	GIS within 2 km radius around the residential address, The distance of the neighbourhood to the nearest park.	Lung cancer incidence	Objective: clinical diagnosis	Green space per capita: null proximity to park: (+)	Proximity to park but not green space per capita was positively associated with lung cancer.	Green space with trees was associated with less incidence of lung cancer.
Wang L 2019 (41) Shanghai	CSS China population Lung cancer: 12, 309 (1063)	Middle aged and older population living at street/town/township administrative unit F: 33% for lung cancer cases 50–86 y	Green space coverage and density at street/town/township level	% of green space coverage to the total area of street/town/township level and how dispersed the green space is using Land Use Status Map	Standardized lung cancer incidence (aged-adjusted)	Objective: hospital record, cases/10,000	Green space coverage: All space units : (-)* Industrial parks excluded: null Green space density: null	Green space at street/town/township level was negatively and significantly associated with lung cancer; however, this association was not significant when the industrial park area was excluded. No association was found between green space density and lung cancer neither.	Not measured
Xie 2018 (47) Hubei	CSS 731(31)	Older population appeared in public space spaces F: 57% 60+ y	Accessibility to park	Nearest distance from the residence to the nearest parks based on the road network	Respiratory diseases	subjective	null	Accessibility to the park did not show any association with the subjectively measured respiratory disease.	Not measured
Zeng 2020a (59) Liaoning	CSS 59,754(0)	School or kindergarten children living in the area for 2+y F: 49% Mean 10.3 ± 3.6 y	Green space around the school	NDVI within 1 km radius around the school (sensitivity analysis with radii of 0.1 km, 0.3 km to 0.5 km)	Current asthma and wheeze	Subjective: ATS-DLD-78-A	Current asthma and wheeze (-)* per 0.1 unit increase in NDVI A dose-response manner relationship was found when analysed in tertiles.	Green space around the school was negatively and significantly associated with current asthma and wheeze in a dose-response manner.	Air pollution exposure and doctor-diagnosed allergy symptoms mediated the association. Physical activity and BMI did not show any mediating effect.

Mortality

Author Year province	Study design Sample size (attrition)	Population (health condition, %female, age)	Environment focus	Exposure/intervention measures and follow ups	Outcomes measured	Method of [outcomes] measurement/ time points measured	Results * p<0.05	Summary of findings	Mediation of effect moderation

Ji 2019 [16] All	CS, pros 31, 618(7, 864)	Oldest-old population from CLHLS cohort F: 62 80+ y, mean 93 ± 7·5 y	Contemporaneous and cumulative residential green space, change in NDVI	NDVI within the 0.25 km and 1.25 km (0.5km as sensitivity analysis) radius around the residential address at the time closest to an event and mean NDVI and change over the follow-up period Follow up: up to 14 y	All-cause mortality except for accidental event	Subjective: from family members	Contemporaneous NDVI0.25, NDVI1.25: (-)*; Cumulative NDVIs: null NDVI change: null	Contemporaneous NDVIs was associated with decreased all-cause mortality. Cumulative NDVIs and change in NDVIs did not show any association with all-cause mortality.	The protective effects of nearby greenness were more pronounced among women and those who exercised.
Ji 2020 [17] All	CS, pros 16, 820(3, 947)	Older population from CLHLS cohort F: 57% 65+ y, mean 87·0 ± 11·3y	Contemporaneous and cumulative residential green Space	NDVI within the 0.5 km radius around the residential address at the time closest to an event and mean NDVI over the follow-up period Follow up: 6 y	All-cause mortality	Subjective: from family members)	Contemporaneous NDVI0.5 (-)* Cumulative NDVIs: null	Contemporaneous NDVI was associated with decreased all-cause mortality. Cumulative NDVI did not show any association.	PM2.5 mediated the association with a small effect size.
Li 2020 [21] All	ES 158 province population	General population F: NA NA y	County level green space coverage	Green space coverage ratio using National Database	Cardiorespiratory mortality (per 100,000 population)	Objective: national database	(-)*	County level green space coverage was negatively and significantly associated with cardiorespiratory mortality.	Not measured
Madaniyazi 2016 [32] All	ES 73 cities population	General population F: NA 19-24 y, mean 21.7 ± 0.4 (SE) y	Green space per capita (moderator) at the city level	Annual green space per capita from Statistical Yearbook	Total, respiratory and cardiovascular mortalities	Objective: national database	All outcomes: (-)*	Annual green space was modifying the negative effect of air pollution on total, respiratory and cardiovascular mortalities.	Not measured
Takano [38] 2002 Shanghai	ES 20 districts	General population F: NA NA y	Green space coverage and urban green space coverage at the district level	% of green space (green areas, park and gardens) or urban green space according to the land use record to the total area or urban area of the district	Mortality (age-adjusted)	Objective: national database (deaths per 1000 population)	Green space coverage years 1995, 1996, 1997: (-)* Urban green space coverage years 1995, 1996, 1997: null	Total green space coverage at the district level but not urban green space coverage was negatively and significantly associated with age-adjusted mortality.	Not measured

Infectious diseases

Author Year province	Study design Sample size (attrition)	Population (health condition, %female, age)	Environment focus	Exposure/intervention measures and follow ups	Outcomes measured	Method of [outcomes] measurement/ time points measured	Results * p<0.05	Summary of findings	Mediation of effect moderation
Chen 2020 [5] Guangdong	ES Guangzhou and Foshan population	General population F: NA NA y	Green space per capita, forest coverage	Statistical Yearbook at a town/street level	Dengue fever	Objective: national database	Green space per capita: (+)* top 10 influencing factors in Guangzhou Forest coverage: (+)* top 8 influencing factors in Foshan	Green space per capita was among the top 10 influencing factors of dengue fever in Guangzhou, and forest coverage was among the top 8 in Foshan.	Not measured
Hundessa 2018 [15] All	ES China population	General population F: NA NA y	Green space at country level	NDVI using US geological survey data	Malaria presence	Objective: national database	Vivax malaria: (+)* falciparum malaria: (+)*	Green space at the country level was significantly associated with vivax malaria and falciparum malaria presence.	Not measured
Liu 2019a [27] All	CS, retro China population	General population F: NA NA y	Green space (including productive plantation and environmental protection area), public green space, green space coverage	Statistics Yearbook of China Follow up: 9 y	Dysentery, tuberculosis, malaria incidence	Objective: national database	**Public green space and green space coverage**: All outcomes: null<br><br>**Green space:** Dysentery: (-)*; tuberculosis, malaria: (+)*	Green space (including productive plantation and environmental protection areas) has a positive association with tuberculosis and malaria incidences and a negative association with dysentery incidence. There was no association between three epidemic diseases and public green space and green space coverage.	Not measured
Zhang 2017 [60] Sichuan, Tibet and Yunnan	ES Hotspots' population	General population F: NA NA y	Forest coverage at the district level	Land cover map	Bacillary dysentery	Objective: national database	non-forest: (+)*	The non-forest area was associated with a risk of bacillary dysentery.	Not measured

Birth outcomes

Author Year province	Study design Sample size (attrition)	Population (health condition, %female, age)	Environment focus	Exposure/intervention measures and follow ups	Outcomes measured	Method of [outcomes] measurement/ time points measured	Results * p<0.05	Summary of findings	Mediation of effect moderation

Lin 2020 [24] Beijing	CS, retro 20, 867(2, 202)	Pregnant women with stillbirth or birth defects, at the gestational age> 43 w F: 100% NR y	Residential green space	Mean NDVI within 0.2 km and 0.5 km radius around participants residential area Follow up: gestation period	Z scores for estimated fetal weight, abdominal circumference, head circumference, femur length and their undergrowth events	Objective: ultrasound	**NDVI 0.25km and 0.5km:** fetal weight, abdominal circumference and head circumference: (+)* when NDVI measured dichotomously (NDVI >median); rest of the outcomes: null	Residential green space predicts z score for estimated fetal weight, abdominal circumference, and head circumference at NDVI 0.5km when measured dichotomously. Sensitivity analyses support the study's main findings.	Air pollution, when it was less than the median estimate, partially mediated the association.
Nie 2020 [33] Guangdong	CCS Case: 8042 Control: 6887	Case: Children with major CHDs Control: Pairs with no major disease F: ~39% 0-1y	Residential green space 1 y prior to the 1st trimester	Mean NDVI within 0.5 km radius around the residential address (sensitivity analysis with a radius of 1 km)	CHDs	Objective: hospital record at birth and one year of follow up	NDVI 0.5km and 1km: 1st QR vs 2nd QR : (-)* 1st QR vs 3rd QR :(-)* 1st QR vs 4th QR :(-)* per IQR of NDVI:(-)*	Maternal residential green pace had a protective effect on the development of CHDs	Air pollutants mediated 52% of associations Effect modification: The protective associations were stronger for fall, urban or permanent residents, higher household income maternal age ≤35 years of age, and high maternal education.
Sun 2020 [37] Guangdong	CCS Case: 2044 Control: 2285	Case: Women with miscarriage in gestational age of less than 28 w Control: healthy pregnant pairs in gestational age of less than 28 w F: 100% Case: mean 30.5 ± 5.5 y Control: mean 28.9 ± 6.0 y	Residential green space (moderator)	NDVI within 1 km radius around the residential area (sensitivity analysis with a radius of 0.5 km)	Miscarriage in gestational age less than 28 w	Objective: hospital record	3rd QR vs 1st QR: ↓* 3rd QR vs 2nd QR: ↓* 3rd QR vs 4th QR: ↓*	The protective effect of green space on the impact of the temperature on the risk of miscarriage was evident in the 3rd QR of green space compared (moderately great green space) to 1st, 2nd and 4th QR at 23.6 C temperature (50th centile).	

Other outcomes

Author Year province	Study design Sample size (attrition)	Population (health condition, %female, age)	Environment focus	Exposure/intervention measures and follow ups	Outcomes measured	Method of [outcomes] measurement/ time points measured	Results * p<0.05	Summary of findings	Mediation of effect moderation
Jia 2016 [74] Zhejiang	RCT, P, 2-arm (1IG, 1CG) 20, n-10 at each group	COPD patients without a current exacerbation F: NR NR y	Forest	Walking in the site for 1.5h twice a day with a 20-min rest during the walk for 7 days	pro-inflammatory cytokines (IL-6, IL-8, TNF-a, CRP, interleukin-1β, interferon-γ) NK, NKT-like, and CD8+ T cells, perforin and granzyme B stress markers (Cortisol, epiendorphine)	Objective: blood test ǀbefore and after	IL-6, IL-8, interferon- γ, interleukin-1b, CRP, CD8+ T-cells, NKT-like cells, Cortisol, epiendorphine: ↓*; TNF-a: ↓; proportions of all lymphocyte subsets, proportions of lymphocyte subsets expressing granzyme, NK cells: (null)	Walking in a forest did decreased pro-inflammatory cytokines, proportions of lymphocyte subsets expressing perforin, stress markers in COPD patients compared to walking in the urban environment.	Not measured
Mao 2012 [78] Zhejiang	RCT, P, 2-arm 20, n=10 at each group	Healthy university students F: 0% Mean 20.8±0.5 y	IG: Forest CG: Urban environment	Walking in the site for 1.5h twice a day with a 20-min rest during the walk for 2 days	IL-6, TNF-a, T-SOD, malondialdehyde, CRP, Total T cell (%), Total B cell(%), Th lymphocyte (%), Ts lymphocyte (%), CD4/CD8, NK cell (%), Platelet activation (CD42a + /CD14), cortisol, testosterone levels	Objective: blood testǀ before and after the test	**Baseline**: all outcomes: null<br><br>**Follow up** TNF-a, IL-6, malondialdehyde, Total B cell, ET-1, cortisol (↓)*; Total B cell↑*; CD4/CD8, NK cell ↑ T-SOD, Th lymphocyte, Ts lymphocyte, platelet activation, testosterone: null	No significant difference between groups at baseline. Walking in a forest significantly decreased pro-inflammatory markers (TNF-a, IL-6), oxidative stress marker (malondialdehyde), cortisol levels, and increased Total B cells compared to walking in the urban environment.	Not measured
Mao 2012a [77] Zhejiang	RCT, p, 2-arm 24(0), IG: 12, CG: 12	Elderly patients with hypertension and CHF class I-III F: NR 60-75 y	IG: Forest CG: Urban environment	Walking in the site for 1.5h twice a day with a 20-min rest during the walk for 7 days	IL-6, TNF-a	Objective: blood testǀ before and after the test	**Baseline**: null<br><br>**Follow up**: null	No significant difference in blood IL-6, TNF-a levels between two groups at baseline and follow-ups.	Not measured

Xie 2018 (47) Hubei	CSS 731(31)	Older population appeared in public space spaces F: 57% 60+ y	Accessibility to park	Nearest distance from the residence to the nearest parks based on the road network	Joint diseases, digestive diseases, endocrine diseases, urological disease, nervous system diseases, respiratory diseases	subjective	Joint diseases and endocrine diseases 3rd QR and 4th QR vs 1st QR: (-)*; digestive diseases, urological diseases, nervous system disease: null	Accessibility to park was associated with a lower odds of joint diseases and endocrine diseases at quartile 3 and 4 compared to the quartile of 1; no association was found for digestive diseases, urological diseases and nervous system disease.	Not measured
Xie 2020 (48) Henan	CSS 27,654(0)	Rural population F: 60% 18–79 y, mean 55.9 ± 12.2 y	Residential green space	NDVI and EVI within 1 km radius around the residential area (sensitivity analysis with radii of 0.5 km and 3 km)	Sleep quality	Subjective: PSQI	per IQR increment in NDVI and EVI: (-)*	Green space was negatively and significantly associated with poor sleep quality.	Air pollution mediated the association. Physical activity and BMI did not show any mediating effect.<br><br>A stronger association was found among males and individuals with higher household income and education.
Zeng 2020b (81) and Lyu 2019b (76) Sichuan	RCT, P, 4-arm (3IG, 1CG) 120(NR), n=30 at each group	Healthy university students F:50% 19-24 y, mean 21.7 ± 0.4 (SE) y	IGs: bamboo forests (YA), bamboo forests (YB), and bamboo park (DJY) CG: urban environment (CS)	3 days of viewing the site in the morning and then walking in the afternoon each for 15 mins	Peripheral oxygen saturation	Objective | before and after the test	**IG1 vs CG:** peripheral oxygen saturation after viewing: ↑*<br><br>**IG2 vs CG**: peripheral oxygen saturation after viewing ↑<br><br>**IG3 vs CG**: peripheral oxygen saturation after viewing ↑	Viewing a bamboo forest or a bamboo park increased peripheral oxygen saturation compared to viewing an urban environment.	Not measured

	60(NR), n=15 at each group	Sub-analysis of male participants	IGs: bamboo forests (YA), bamboo forests (YB), and bamboo park (DJY) CG: urban environment (CS)	3 days of viewing the site in the morning and then walking in the afternoon each for 15 mins	Peripheral oxygen saturation, NK activity number of NK cells, perforin, granulysin, granzyme A, granzyme B, corticosterone	Objective	Corticosterone ↓* NK activity, number of NK cells, perforin, granzyme A, granzyme B ↑*; peripheral oxygen saturation, granulysin: null	All types of exposure to bamboo forest or park significantly decreased SBP, mood disturbance, and its negative subcomponents, and increased immune system-related biomarkers compared to the control group	Not measured
Zhu 2019b-study 1 [(67)] All	CS, pros 39, 225 (19,400)	Older population from CLHLS cohort F: 59% 65+ y, mean 88±11.5 y	Residential green space	Baseline mean NDVI within 0.5km radius around the residential addresses Follow up: 2-14 y	Cognitive function and impairment	Subjective: MMSE, and MMSE <24	Cognitive function and impairment: null	Residential green space did not predict cognitive function among the older population.	Not measured
Zhu 2019b-study 2 [(67)] All	CSS 39, 225(898)	Older population from CLHLS cohort F: 59% 65+ y, mean 88±11.5 y	Residential green space	Baseline mean NDVI within 0.5km radius around the residential addresses	Cognitive function and impairment	Subjective: MMSE, and MMSE <25	cognitive function and lack of cognitive impairment: (+)*	Residential green space was positively and significantly associated with cognitive function and lack of cognitive impairment.	Not measured
Zhu 2020a [(68)] All	CS, pros Total: 19,726 (12, 731) 65-79 y: 3470 80+ y: 3524	Older population from CLHLS cohort F: 51% 65+ y, mean 80.0 ± 11.0 y	Residential green space	Baseline mean NDVI within 0.5 km radius around the residential addresses Follow up: 2-14 y	Cognitive function	Subjective: MMSE	All age: 1st QR vs 4th QR and 0.1-unit of NDVI (+)* 65-79 y: 1st QR vs 4th QR and 0.1-unit of NDVI (+)* 80+: null	Residential green space was positively and significantly associated with cognitive function for analysis of all ages and those aged 65 - 79 y but not for those aged 80+ y	The protective effect of residential greenness on cognitive impairment was observed among the non-ε4 carriers but not among the ε4 carriers; the interaction was insignificant.
Zhu 2020c [(70)] All	CS, pros 2, 439(1, 103)	Older population +65 from CLHLS cohort F: 53% Mean 83 ± 11.7 y	Residential green space	Baseline mean NDVI within 0.5 km radius around the residential addresses Follow up: 12 y	Vitamin D deficiency Vitamin D (change)	Objective: blood test	0.1-unit increase in NDVI, stable vs improved: (+)*	Residential green space was positively and significantly associated with vitamin D non-deficiency and improved the level of vitamin D	The association was stronger in men and people free from disability at baseline.

Int. J. Environ. Res. Public Health **2021**, 18, 9937

Abbreviations: ADHD, attention deficient hyperactivity disorder; ADL, disabilities in activities of daily living; Ang II, angiotensin II; AT1, angiotensin II type 1 receptor; AT2, angiotensin II type 2 receptor; ATS-DLD-78-A, standardization project questionnaire of the American Thoracic Society; BAS, before-after study; BMI, Body mass index; BNP, brain natriuretic peptide; CBCL; childhood behavior checklist; CCS, case-control study; CES-D, epidemiologic studies depression scale; CG, control group; CHD, congenital heart defect; CHF, chronic heart failure; CO, cross-over; COPD, chronic obstructive pulmonary disease; CS-pros; cohort study-prospective; CS-retro, cohort study-retrospective; CSS, cross-sectional study; DBP, diastolic blood pressure; DSM-IV, diagnostic and statistical manual of mental disorders-Fourth Edition; EEG, electroencephalogram; EQLS, European quality of life survey; ET-1, edothelin-1; EVI, Enhanced vegetation index; FI, frailty index; GHQ-12, general health questionnaire; GIS, geographic information system; HDL-C, high-density lipoprotein cholesterol; HOMA-IR, homeostatic model assessment-insulin resistance; HOMA-B, homeostatic model assessment-β-cell function; HRV, heart rate variability; IADL, instrumental activities of daily living, IG, intervention group, IL-6, interleukin-6; IL-8, interleukin-8; ISAAC, asthma and allergies in childhood questionnaire; LDL-C, low-density lipoprotein cholesterol; MHSs, national mental health services survey; MMSE, mini-mental state examination; NDVI, normalized difference vegetation index; NT-ProBNP, N-terminal-pro hormone brain natriuretic peptide; P, parallel; PoMS, profile of mood states, PSQI, Pittsburgh sleep quality index; QoL, quality of life; QR, interquartile range; RAS, renin–angiotensin system; RCT, Randomised controlled trial; ROS, restorative outcome scale; SAVI, soil adjusted; vegetation index; SBP, systolic blood pressure; SDM, semantic differential method; SF, short form health survey 12-Item or 36- Item; SSL-I, social well-being scale and social support list-interactions; STAI; State-trait anxiety inventory; SVS; Subjective Vitality Scale; SWBS, subjective well being scale; TC, total cholesterol; TG, triglycerides; TNF-a, tumour necrosis factor-a; T-SOD, total super oxide dismutase; VAS, visual analogue scale; VCF, vegetation continuous field; WC, waist circumfer- ence; WHOQOL-BREF, WHO quality of life assessment brief version; WHO- 5, World Health Organization well-being index; (+), positive association; (-), negative association; ↑, increase; ↓, decrease.

References

1. Bao WW, Yang BY, Zou ZY, *et al.* Greenness surrounding schools and adiposity in children and adolescents: Findings from a national population-based study in China. *Environmental Research*. 2021;192:110289.
2. Chang P-J, Lin Y, Song R, *et al.* Leisure satisfaction mediates the relationships between leisure settings, subjective well-being, and depression among middle-aged adults in Urban China. *Applied Research in Quality of Life*. 2019;14(4):1001-17.
3. Chen H, Liu Y, Zhu Z, *et al.* Does where you live matter to your health? Investigating factors that influence the self-rated health of urban and rural Chinese residents: evidence drawn from Chinese General Social Survey data. *Health Qual Life Outcomes*. 2017;15(1):78.
4. Chen Y, Stephens M, Jones CA. Does Residents' Satisfaction with the Neighbourhood Environment Relate to Residents' Self-Rated Health? Evidence from Beijing. *Int J Environ Res Public Health*. 2019;16(24).
5. Chen Y, Yang Z, Jing Q, *et al.* Effects of natural and socioeconomic factors on dengue transmission in two cities of China from 2006 to 2017. *Science of the Total Environment*. 2020;724:138200.
6. Dong H, Qin B. Exploring the link between neighborhood environment and mental wellbeing: A case study in Beijing, China. *Landscape and Urban Planning*. 2017;164:71-80.
7. Fan S, Xue Z, Yuan J, *et al.* Associations of residential greenness with diabetes mellitus in chinese uyghur adults. *International Journal of Environmental Research and Public Health*. 2019;16(24):5131.
8. Fan S, Yang BY, Xue Z, *et al.* Associations between residential greenness and blood lipids in Chinese Uyghur adults. *Environment International*. 2020;142:105903.
9. Fan J, Guo Y, Cao Z, *et al.* Neighborhood greenness associated with chronic obstructive pulmonary disease: A nationwide cross-sectional study in China. *Environmental International*. 2020;144:106042.
10. Helbich M, Yao Y, Liu Y, *et al.* Using deep learning to examine street view green and blue spaces and their associations with geriatric depression in Beijing, China. *Environmental International*. 2019;126:107-17.
11. Huang B, Liu Y, Feng Z, *et al.* Residential exposure to natural outdoor environments and general health among older adults in Shanghai, China. *International Journal for Equity in Health*. 2019;18(1):N.PAG-N.PAG.
12. Huang B, Huang C, Feng Z, *et al.* Association between residential greenness and general health among older adults in rural and urban areas in China. *Urban Forestry and Urban Greening*. 2020.
13. Huang B, Liu Y, Chen Y, *et al.* Establishing associations between residential greenness and markers of adiposity among middle-aged and older Chinese adults through multilevel structural equation models. *International journal of hygiene and environmental health*. 2020;230:113606.
14. Huang W-Z, Yang B-Y, Yu H-Y, *et al.* Association between community greenness and obesity in urban-dwelling Chinese adults. *The Science of the total environment*. 2020;702:135040.
15. Hundessa S, Li S, Liu L, *et al.* Projecting environmental suitable areas for malaria transmission in China under climate change scenarios. *Environ Res*. 2018;162:203-10.
16. Ji JS, Zhu A, Bai C, *et al.* Residential greenness and mortality in oldest-old women and men in China: a longitudinal cohort study. *The Lancet Planetary health*. 2019;3(1):e17-e25.
17. Ji JS, Zhu A, Lv Y, *et al.* Interaction between residential greenness and air pollution mortality: analysis of the Chinese Longitudinal Healthy Longevity Survey. *The Lancet Planetary Health*. 2020;4(3):e107-e15.
18. Jia X, Yu Y, Xia W, *et al.* Cardiovascular diseases in middle aged and older adults in China: the joint effects and mediation of different types of physical exercise and neighborhood greenness and walkability. *Environmental Research*. 2018;167:175-83.
19. Leng H, Li S, Yan S, *et al.* Exploring the relationship between green space in a neighbourhood and cardiovascular health in the winter city of China: A study using a health survey for harbin. *International Journal of Environmental Research and Public Health*. 2020;17(2):513.
20. Li L, Hart JE, Coull BA, *et al.* Effect of Residential Greenness and Nearby Parks on Respiratory and Allergic Diseases among Middle School Adolescents in a Chinese City. *International Journal of Environmental Research and Public Health*. 2019;16(6).
21. Li C, Song Y, Tian L, *et al.* Urban form, air quality, and cardiorespiratory mortality: A path analysis. *International Journal of Environmental Research and Public Health*. 2020;17(4):1202.
22. Liao J, Chen X, Xu S, *et al.* Effect of residential exposure to green space on maternal blood glucose levels, impaired glucose tolerance, and gestational diabetes mellitus. *Environmental Research*. 2019;176:108526.
23. Liao J, Yang S, Xia W, *et al.* Associations of exposure to green space with problem behaviours in preschool-aged children. *International journal of epidemiology*. 2020;49(3):944-53.
24. Lin L, Li Q, Yang J, *et al.* The associations of residential greenness with fetal growth in utero and birth weight: A birth cohort study in Beijing, China. *Environment International*. 2020;141:105793.
25. Liu M, Huang Y, Hiscock R, *et al.* Do Climate Change Policies Promote or Conflict with Subjective Wellbeing: A Case Study of Suzhou, China. *International Journal of Environmental Research and Public Health*. 2016;13(3):344.
26. Liu H, Li F, Li J, *et al.* The relationships between urban parks, residents' physical activity, and mental health benefits: A case study from Beijing, China. *J Environ Manage*. 2017;190:223-30.
27. Liu L, Zhong Y, Ao S, *et al.* Exploring the relevance of green space and epidemic diseases based on panel data in China from 2007 to 2016. *International Journal of Environmental Research and Public Health*. 2019;16(14):2551.

28. Liu Y, Wang R, Xiao Y, *et al.* Exploring the linkage between greenness exposure and depression among Chinese people: Mediating roles of physical activity, stress and social cohesion and moderating role of urbanicity. *Health & Place*. 2019;58:N.PAG-N.PAG.
29. Liu Y, Wang R, Grekousis G, *et al.* Neighbourhood greenness and mental wellbeing in Guangzhou, China: What are the pathways? *Landscape and Urban Planning*. 2019;190.
30. Liu Y, Wang R, Lu Y, *et al.* Natural outdoor environment, neighbourhood social cohesion and mental health: Using multilevel structural equation modelling, streetscape and remote-sensing metrics. *Urban Forestry and Urban Greening*. 2020;48.
31. Lu T, Lane M, Van der Horst D, *et al.* Exploring the impacts of living in a "green" city on individual BMI: A study of Lingang new town in Shanghai, China. *International Journal of Environmental Research and Public Health*. 2020;17(19):1-15.
32. Madaniyazi L, Guo Y, Chen R, *et al.* Predicting exposure-response associations of ambient particulate matter with mortality in 73 Chinese cities. *Environmental pollution (Barking, Essex : 1987)*. 2016;208(Pt A):40-7.
33. Nie Z, Yang B, Ou Y, *et al.* Maternal residential greenness and congenital heart defects in infants: A large case-control study in Southern China. *Environment International*. 2020;142:105859.
34. Peng W, Jiang M, Shi H, *et al.* Cross-sectional association of residential greenness exposure with activities of daily living disability among urban elderly in Shanghai. *International journal of hygiene and environmental health*. 2020;230:113620.
35. Qiu Y, Liu Y, Li Z. Exploring the linkage between the neighborhood environment and mental health in Guangzhou, China. *International Journal of Environmental Research and Public Health*. 2019;16(17):3206.
36. Qu Y, Yang B, Lin S, *et al.* Associations of greenness with gestational diabetes mellitus: The Guangdong Registry of Congenital Heart Disease (GRCHD) study. *Environmental Pollution*. 2020;266:115127.
37. Sun X, Luo X, Cao G, *et al.* Associations of ambient temperature exposure during pregnancy with the risk of miscarriage and the modification effects of greenness in Guangdong, China. *Science of the Total Environment*. 2020;702:134988.
38. Takano T, Fu J, Nakamura K, *et al.* Age-adjusted mortality and its association to variations in urban conditions in Shanghai. *Health Policy*. 2002;61(3):239-53.
39. Tang S, Lee HF, Feng J. Social capital, built environment and mental health: A comparison between the local elderly people and the ' laopiao ' in urban China. *Ageing and Society*. 2020:1-25.
40. Wang L, Zhao X, Xu W, *et al.* Correlation analysis of lung cancer and urban spatial factor: Based on survey in Shanghai. *Journal of Thoracic Disease*. 2016;8(9):2626-37.
41. Wang L, Sun W, Zhou K, *et al.* Spatial analysis of built environment risk for respiratory health and its implication for urban planning: A case study of shanghai. *International Journal of Environmental Research and Public Health*. 2019;16(8):1455.
42. Wang R, Helbich M, Yao Y, *et al.* Urban greenery and mental wellbeing in adults: Cross-sectional mediation analyses on multiple pathways across different greenery measures. *Environmental Research*. 2019;176:108535.
43. Wang R, Liu Y, Xue D, *et al.* Depressive symptoms among Chinese residents: how are the natural, built, and social environments correlated? *BMC Public Health*. 2019;19(1):887.
44. Wang R, Yang B, Yao Y, *et al.* Residential greenness, air pollution and psychological well-being among urban residents in Guangzhou, China. *Science of the Total Environment*. 2020;711:134843.
45. Wang Z, Zhang C, Chen Y, *et al.* The effects of urban park on residents' health: An empirical study based on Tangxi River Park in Hefei. *IOP Conference Series: Earth and Environmental Science*. 2020;569.
46. Xiao X, Yang B-Y, Hu L-W, *et al.* Greenness around schools associated with lower risk of hypertension among children: Findings from the Seven Northeastern Cities Study in China. *Environmental pollution (Barking, Essex : 1987)*. 2020;256:113422.
47. Xie B, An Z, Zheng Y, *et al.* Healthy aging with parks: Association between park accessibility and the health status of older adults in urban China. *Sustainable Cities and Society*. 2018;43:476-86.
48. Xie Y, Xiang H, Di N, *et al.* Association between residential greenness and sleep quality in Chinese rural population. *Environmental International*. 2020;145:106100.
49. Xue T, Zhu T, Zheng Y, *et al.* Declines in mental health associated with air pollution and temperature variability in China. *Nature Communications*. 2019;10(1):2165.
50. Yang B-Y, Markevych I, Bloom MS, *et al.* Community greenness, blood pressure, and hypertension in urban dwellers: The 33 Communities Chinese Health Study. *Environment International*. 2019;126:727-34.
51. Yang B-Y, Markevych I, Heinrich J, *et al.* Associations of greenness with diabetes mellitus and glucose-homeostasis markers: The 33 Communities Chinese Health Study. *International Journal of Hygiene & Environmental Health*. 2019;222(2):283-90.
52. Yang B-Y, Markevych I, Heinrich J, *et al.* Residential greenness and blood lipids in urban-dwelling adults: The 33 Communities Chinese Health Study. *Environmental pollution (Barking, Essex : 1987)*. 2019;250:14-22.
53. Yang BY, Zeng XW, Markevych I, *et al.* Association between Greenness Surrounding Schools and Kindergartens and Attention-Deficit/Hyperactivity Disorder in Children in China. *JAMA Network Open*. 2019;2(12):190673.
54. Yang M, Dijst M, Faber J, *et al.* Using structural equation modeling to examine pathways between perceived residential green space and mental health among internal migrants in China. *Environmental Research*. 2020;183:109121.
55. Yang B-Y, Liu K-K, Markevych I, *et al.* Association between residential greenness and metabolic syndrome in Chinese adults. *Environment International*. 2020;135:105388.
56. Yang B-Y, Hu L-W, Jalaludin B, *et al.* Association Between Residential Greenness, Cardiometabolic Disorders, and Cardiovascular Disease Among Adults in China. *JAMA Network Open*. 2020;3(9):e2017507-e.

57. Yang T, Barnett R, Fan Y, *et al.* The effect of urban green space on uncertainty stress and life stress: A nationwide study of university students in China. *Health & Place*. 2019;59:N.PAG-N.PAG.
58. Ying Z, Ning LD, Xin L. Relationship between built environment, physical activity, adiposity, and health in adults aged 46-80 in Shanghai, China. *Journal of Physical Activity and Health*. 2015;12(4):569-78.
59. Zeng XW, Lowe AJ, Lodge CJ, *et al.* Greenness surrounding schools is associated with lower risk of asthma in schoolchildren. *Environment International*. 2020;143:105967.
60. Zhang H, Si Y, Wang X, *et al.* Environmental Drivers and Predicted Risk of Bacillary Dysentery in Southwest China. *Int J Environ Res Public Health*. 2017;14(7).
61. Zhang L, Kwan MP, Chen F, *et al.* Impacts of individual daily greenspace exposure on health based on individual activity space and structural equation modeling. *International Journal of Environmental Research and Public Health*. 2018;15(10):2323.
62. Zhang CJP, Barnett A, Johnston JM, *et al.* Objectively-measured neighbourhood attributes as correlates and moderators of quality of life in older adults with different living arrangements: The alecs cross-sectional study. *International Journal of Environmental Research and Public Health*. 2019;16(5).
63. Zhao Y, Yu F, Jing B, *et al.* An analysis of well-being determinants at the city level in China using big data. *Social Indicators Research*. 2019;143(3):973-94.
64. Zhou M, Tan S, Tao Y, *et al.* Neighborhood socioeconomics, food environment and land use determinants of public health: Isolating the relative importance for essential policy insights. *Land Use Policy*. 2017;68:246-53.
65. Zhou Y, Yuan Y, Chen Y, *et al.* Association Pathways Between Neighborhood Greenspaces and the Physical and Mental Health of Older Adults—A Cross-Sectional Study in Guangzhou, China. *Frontiers in Public Health*. 2020;8.
66. Zhu A, Yan LL, Wu CD, *et al.* Residential greenness, activities of daily living, and instrumental activities of daily living: A longitudinal cohort study of older adults in China. *Environmental Epidemiology*. 2019;3(5):e065.
67. Zhu A, Wu C, Yan LL, *et al.* Association between residential greenness and cognitive function: analysis of the Chinese Longitudinal Healthy Longevity Survey. *BMJ Nutrition, Prevention & Health*. 2019:bmjnph-2019-000030.
68. Zhu A, Yan L, Shu C, *et al.* APOE epsilon4 Modifies Effect of Residential Greenness on Cognitive Function among Older Adults: A Longitudinal Analysis in China. *Scientific reports*. 2020;10(1):82.
69. Zhu A, Yan L, Wu C, *et al.* Residential Greenness and Frailty Among Older Adults: A Longitudinal Cohort in China. *Journal of the American Medical Directors Association*. 2020;21(6):759-.
70. Zhu A, Zeng Y, Ji JS. Residential Greenness Alters Serum 25(OH)D Concentrations: A Longitudinal Cohort of Chinese Older Adults. *Journal of the American Medical Directors Association*. 2020.
71. Elsadek M, Liu B, Lian Z. Green façades: Their contribution to stress recovery and well-being in high-density cities. *Urban Forestry and Urban Greening*. 2019;46.
72. Elsadek M, Liu B, Lian Z, *et al.* The influence of urban roadside trees and their physical environment on stress relief measures: A field experiment in Shanghai. *Urban Forestry & Urban Greening*. 2019;42:51-60.
73. Hassan A, Tao J, Li G, *et al.* Effects of Walking in Bamboo Forest and City Environments on Brainwave Activity in Young Adults. *Evid Based Complement Alternat Med*. 2018;2018:9653857.
74. Jia BB, Yang ZX, Mao GX, *et al.* Health effect of forest bathing trip on elderly patients with chronic obstructive pulmonary disease. *Biomedical and Environmental Sciences*. 2016;29(3):212-8.
75. Lyu B, Zeng C, Deng S, *et al.* Bamboo forest therapy contributes to the regulation of psychological responses. *Journal of Forest Research*. 2019;24(1):61-70.
76. Lyu B, Zeng C, Xie S, *et al.* Benefits of a three-day bamboo forest therapy session on the psychophysiology and immune system responses of male college students. *International journal of environmental research and public health*. 2019;16(24):4991.
77. Mao GX, Cao YB, Lan XG, *et al.* Therapeutic effect of forest bathing on human hypertension in the elderly. *Journal of Cardiology*. 2012;60(6):495-502.
78. Mao GX, Lan XG, Cao YB, *et al.* Effects of short-term forest bathing on human health in a broad-leaved evergreen forest in Zhejiang Province, China. *Biomedical and Environmental Sciences*. 2012;25(3):317-24.
79. Mao GX, Lan XG, Chen ZM, *et al.* The salutary influence of forest bathing on elderly patients with chronic heart failure. *Journal of the American Geriatrics Society*. 2017;64(Supplement 2):S357-S8.
80. Wu Q, Cao Y, Mao G, *et al.* Effects of forest bathing on plasma endothelin-1 in elderly patients with chronic heart failure: Implications for adjunctive therapy. *Geriatrics and Gerontology International*. 2017;17(12):2627-9.
81. Zeng C, Lyu B, Deng S, *et al.* Benefits of a three-day bamboo forest therapy session on the physiological responses of university students. *International Journal of Environmental Research and Public Health*. 2020;17(9).
82. Li D, Zhai Y, Xiao Y, *et al.* Subtypes of park use and self-reported psychological benefits among older adults: A multilevel latent class analysis approach. *Landscape and Urban Planning*. 2019;190.
83. Zhou C, Yan L, Yu L, *et al.* Effect of Short-term Forest Bathing in Urban Parks on Perceived Anxiety of Young-adults: A PilotStudy in Guiyang, Southwest China. *Chinese Geographical Science*. 2019;29(1):139-50.
